# Phylogeny of the Neotropical Hypoctonine Whip-Scorpions (Thelyphonida, Thelyphonidae), with Descriptions of Two New Genera and Species †

**DOI:** 10.3390/insects15100761

**Published:** 2024-09-30

**Authors:** Ricardo Botero-Trujillo, Jairo A. Moreno-González, Lorenzo Prendini

**Affiliations:** Division of Invertebrate Zoology, American Museum of Natural History, Central Park West at 79th Street, New York, NY 10024-5192, USA; jmorenogonzalez@amnh.org (J.A.M.-G.); lorenzo@amnh.org (L.P.)

**Keywords:** Uropygi, neotropics, systematics, taxonomy, vinegaroons, Colombia, Venezuela

## Abstract

**Simple Summary:**

Thelyphonida, also known as vinegaroons or whip-scorpions, is a small arachnid order with 140 described species. Despite being conspicuous and widely distributed across the tropics and subtropics on four continents, knowledge of the order has been slow to advance. The genus *Thelyphonellus*, a member of subfamily Hypoctoninae, consisted of four species described prior to the present contribution. In this study, the first detailed morphological study and phylogenetic analysis of *Thelyphonellus* is presented. The analysis includes all except one of the previously described species of *Thelyphonellus* in addition to two new species described herein, the species of *Ravilops* (from the Caribbean island of Hispaniola), and the West African genus *Etienneus*. A single, optimal phylogenetic hypothesis placed *Ravilops* nested within *Thelyphonellus*. Four lineages with disjunct distributions, each characterized by a combination of characters, were recovered by the analysis of Hypoctoninae. Based on these results, four genera, two of which are new, are recognized: *Ravilops*, *Thelyphonellus*, *Wounaan* (new genus), and *Yekuana* (new genus). Two new species, classified in *Wounaan* and *Yekuana*, are described and illustrated. A key to the identification of the Neotropical genera of Hypoctoninae and a map plotting the known distribution of its species are also presented.

**Abstract:**

Thelyphonida Blanchard, 1852, also known as vinegaroons or whip-scorpions, is a small arachnid order with 140 described species contained in a single family, Thelyphonidae Lucas, 1835. Despite being conspicuous and widely distributed across the tropics and subtropics on four continents, knowledge of the order has been slow to advance. Hypoctoninae Pocock, 1899, one of four subfamilies currently recognized and one of two represented in the New World, comprises five genera. Since its inception, *Thelyphonellus* Pocock, 1894 has remained the only hypoctonine genus occurring in South America, with only four species described prior to the present contribution. The first detailed morphological study and phylogenetic analysis of *Thelyphonellus* is presented herein. The morphological phylogenetic analysis—the first for Thelyphonida—includes all except one of the previously described species of *Thelyphonellus* in addition to two new species described herein; the species of *Ravilops* Víquez and Armas, 2005 (from the Caribbean island of Hispaniola); and the monotypic Old World genus *Etienneus* Heurtault, 1984 (from West Africa) scored for 45 morphological characters. A single, most parsimonious phylogenetic hypothesis revealed that *Thelyphonellus* is paraphyletic with respect to *Ravilops*. The New World Hypoctoninae comprises four clades with disjunct distributions and well supported by a combination of morphological characteristics, on the basis of which four genera, two of which are new, are recognized: *Ravilops*, with two species, endemic to Hispaniola; *Thelyphonellus*, herein restricted to *Thelyphonellus amazonicus* (Butler, 1872) and *Thelyphonellus ruschii* Weygoldt, 1979, occurring in Guyana, Suriname, French Guiana, and northern Brazil; *Wounaan*, **gen. n.**, containing *Wounaan vanegasae* (Giupponi and Vasconcelos, 2008), **comb. n.** and *Wounaan yarigui*, **sp. n.** from Colombia; and *Yekuana*, **gen. n.**, containing *Yekuana venezolensis* (Haupt, 2009), **comb. n.** and *Yekuana wanadi*, **sp. n.** from Venezuela. The two new species are described and illustrated. A key to the identification of the Neotropical genera of Hypoctoninae and a map plotting the known distribution of its species are also presented.

## 1. Introduction

Thelyphonida Blanchard, 1852, commonly known as vinegaroons or whip-scorpions, is an order of circumtropical arachnids easily recognizable by their robust, horizontally oriented raptorial pedipalps; terminal multisegmented flagellum; and repugnatorial glands on the last abdominal segment used for expelling noxious defense secretions [1,2]. Together with the short-tailed whip-scorpion order Schizomida Petrunkevitch, 1945, Thelyphonida forms a monophyletic group known as Uropygi Thorell, 1883 [3,4,5,6,7]. A single family of whip-scorpions, Thelyphonidae Lucas, 1835, with 25 genera (16 extant and 9 fossil) and 140 species (126 extant and 14 fossil), is currently recognized [8].

Despite being conspicuous and widely distributed in the rainforests and savannas of the tropics and subtropics on four continents (North America, South America, Africa, and Asia), knowledge of this arachnid order has been slow to advance. Although originally comprising a single monotypic genus, *Thelyphonus* Latreille, 1802, many new species and at least nine new genera were added to the order during the 19th century (i.e., [9,10,11,12,13,14,15,16,17,18,19,20,21,22,23,24,25,26,27,28,29]), and some species of *Thelyphonus* were transferred or synonymized [24,28,29,30]. Five new genera and additional species were added in the late 19th century and early 20th century, when the first suprageneric classifications of the order were also proposed [31,32,33,34,35,36,37,38,39,40,41,42,43,44,45].

Pocock (1899) [46] suggested that the order should be divided into two subfamilies: Thelyphoninae Lucas, 1835 (as Thelyphonini) for genera with a carina between the median and lateral ocelli and Hypoctoninae Pocock, 1899 (as Hypoctonini) for genera without the carina. Gravely (1916) [37] further separated the genera with a carapacial carina into three main groups based on the sexual dimorphism of the male pedipalp patellar apophysis. Later, Speijer (1933) [44] proposed the family Mastigoproctidae Speijer, 1933 for *Mastigoproctus* Pocock, 1894, to which *Teltus* Speijer, 1936 was added, arguing that these taxa possess eight tarsal segments on leg I instead of seven, as in Thelyphonidae [45]. Rowland and Cooke (1973) [47] provided a comprehensive generic and suprageneric revision of the order, elevating Hypoctoninae to the rank of family (Hypoctonidae) and creating two new as subfamilies of Thelyphonidae: Typopeltinae Rowland and Cooke, 1973 and Uroproctinae Rowland and Cooke, 1973. However, Weygoldt (1979) [48] argued that the Rowland and Cooke’s (1973) [47] classification was unsupported by phylogenetic evidence. Following these criticisms, Hypoctonidae and Mastigoproctidae were returned to the rank of subfamily as Hypoctoninae and Mastigoproctinae, respectively [49], and Uroproctinae was synonymized with Mastigoproctinae [50], resulting in four subfamilies within Thelyphonidae: Hypoctoninae, Mastigoproctinae, Thelyphoninae, and Typopeltinae [8]. 

The widely distributed subfamily Hypoctoninae comprises five genera: *Etienneus* Heurtault, 1984 (Africa: Burkina Faso, Guinea Bissau, Senegal, and The Gambia); *Hypoctonus* Thorell, 1888 (Asia: Bangladesh, Bhutan, China, India, Indonesia, Myanmar, and Thailand); *Labochirus* Pocock, 1894 (Asia: India and Sri Lanka); *Ravilops* Víquez and Armas, 2005 (Antilles: Hispaniola: Dominican Republic, and Haiti); and *Thelyphonellus* Pocock, 1894 (South America: Brazil, Colombia, French Guiana, Guyana, Suriname, and Venezuela). This subfamily was recorded for the first time in the New World, with the description of *Thelyphonus amazonicus* Butler, 1872 from Santarém, Alter do Chão, in the Brazilian state of Pará [19]. Subsequently, Pocock (1894) [28] created *Thelyphonellus*, defined by the absence of ommatoids on the last opisthosomal segment and the carapace acarinate and pointed anteriorly, and designated *Thelyphonellus amazonicus* (Butler, 1872) as its type species. More than 80 years later, Weygoldt (1979) [48] redescribed *T. amazonicus* and described a second species in the genus, *Thelyphonellus ruschii* Weygoldt, 1979, based on specimens from Demerara, Guyana. 

Armas (2002) [51] described *Thelyphonellus wetherbeei* Armas, 2002 from the Parque Nacional Armando Bermúdez in the Santiago Province of the Dominican Republic, which Víquez and Armas (2005) [52] transferred to a new monotypic genus, *Ravilops* Víquez and Armas, 2005, i.e., *Ravilops wetherbeei* (Armas, 2002), based on the female spermathecae. Giupponi and Vasconcelos (2008) [53] subsequently described *Thelyphonellus vanegasae* Giupponi and Vasconcelos, 2008 from Dagua in the Valle del Cauca Department of Colombia, and Haupt (2009) [54] described *Thelyphonellus venezolanus* Haupt, 2009 from San Isidro in the Bolívar State of Venezuela. Teruel (2017) [55] described a second species of *Ravilops*, *Ravilops kovariki* Teruel, 2017, based on specimens from Neiba in the Bahoruco Province of the Dominican Republic. None of these contributions to the knowledge of New World Hypoctoninae tested the monophyly of *Thelyphonellus* or the validity of *Ravilops* in a phylogenetic framework, however. Aside from a detailed redescription of the African species *Etienneus africanus* (Hentschel, 1899) by Huff and Prendini (2009) [56] and the inclusion of two hypoctonine exemplar species, *E. africanus* and *T. amazonicus*, in a molecular phylogeny of Uropygi [57], the phylogeny and morphology of Hypoctoninae has never been explored. 

The present contribution provides the first phylogenetic analysis of Hypoctoninae whip-scorpions, focusing on the New World taxa and based on a matrix of 45 morphological characteristics. A single, most parsimonious phylogenetic hypothesis revealed that *Thelyphonellus* is paraphyletic with respect to *Ravilops*. The New World Hypoctoninae comprises four clades with disjunct distributions and well supported by a combination of discrete morphological characteristics, on the basis of which four genera, two of which are new, are recognized. Two new species are described and illustrated. A key to the identification of the Neotropical genera of Hypoctoninae and a map plotting the known distribution of its species are also presented.

## 2. Materials and Methods

### 2.1. Material and Taxon Sampling

Ten species of Thelyphonidae were examined for the present contribution. One, *Ginosigma schimkewitschi* (Tarnani, 1894), belongs to subfamily Thelyphoninae, whereas the others belong to Hypoctoninae: *E. africanus*, *R. kovariki*, *R. wetherbeei*, *T. amazonicus*, *T.* aff. *ruschii*, *W. vanegasae*, *W. yarigui*, *Y. venezolensis*, and *Y. wanadi*.

Material was deposited in the following collections: the American Museum of Natural History (AMNH), including the Ambrose Monell Cryocollection for Molecular and Microbial Research (AMCC), New York, USA; the Instituto de Investigación de Recursos Biológicos “Alexander von Humboldt” (IAvH), Villa de Leyva, Colombia; the Museo Departamental de Ciencias Naturales “Federico Carlos Lehmann Valencia” (IMCN), Instituto para la Investigación y la Preservación del Patrimonio Cultural y Natural del Valle del Cauca, Cali, Colombia; the Museu de Zoologia, Universidade de São Paulo (MZSP), Brazil; the Museu Paraense Emílio Goeldi (MPEG), Belém, Brazil; the Museum für Naturkunde der Humboldt-Universität, Berlin, Germany (ZMB); the Museum of Comparative Zoology (MCZ), Harvard University, Cambridge, MA, USA; the Natural History Museum, London, UK (BMNH); the Senckenberg Forschungsinstitut und Naturmuseum, Frankfurt (SMF), Germany; and the U.S. National Museum of Natural History (USNM), Smithsonian Institution, Washington, DC, USA. 

### 2.2. Descriptions and Terminology

The description of the two new species follows the standard set by Huff and Prendini’s (2009) [56] redescription of *E. africanus*, except that the positional descriptors “internal” and “external” were replaced with “prolateral” and “retrolateral.” Nomenclature for the male gonopods follows Seraphim et al. (2019) [58].

### 2.3. Microscopy, Measurement, and Imaging

Specimens were examined with a Nikon SMZ 1500 stereomicroscope (Nikon, Tokyo, Japan), equipped with a calibrated ocular micrometer for measurements. Measurements follow Barrales-Alcalá et al. (2018) [59] with the addition of some other structures. Photographs were taken with a Nikon DS-Ri2 camera (Nikon, Tokyo, Japan), adapted to a Nikon SMZ 18 stereomicroscope (Nikon, Tokyo, Japan) with a SHR Plan Apo 1× Objective, using NIS-Elements Imaging Software, ver. 4.60, at the AMNH Microscopy and Imaging Facility. Focused images were edited with GIMP 2.10 (http://www.gimp.org/, accessed on 10 June 2024) and plates created using Inkscape 1.2.2 (http://www.inkscape.org/, accessed on 20 May 2024).

### 2.4. Georeferencing and Mapping

The two-dimensional distribution map was produced with QGIS Geographic Information System 3.30 (http://www.qgis.org/, accessed on 20 May 2024) using a digital elevation model (DEM), raster Hillshade conversion (with layers on azimuths 45° and 145°), and single-band rendering (i.e., BrBg). Georeferences for plotted localities were extracted from the original collection data of existing specimens, the World Uropygi Catalog (2024) [8], or acquired retroactively using the GeoNames Server (http://www.geonames.org/, accessed on 20 May 2024).

### 2.5. Phylogenetic Analysis

A matrix of 45 new morphological characteristics (32 binary and 13 multistate), scored for ten species in six genera, was constructed (Appendix A, Appendix B, Appendix C) and deposited in MorphoBank (Project 5288: Phylogeny of the Neotropical Hypoctonine Whip-scorpions). The ingroup comprises nine species of subfamily Hypoctoninae: four species formerly placed in *Thelyphonellus*, from South America (*T. amazonicus*, *T. vanegasae*, *T. venezolanus*, and *T.* aff. *ruschii*); two species of *Ravilops*, from the Dominican Republic on the Caribbean island of Hispaniola (*R. kovariki* and *R. wetherbeei*); two new species, described herein and assigned to new genera, from Colombia and Venezuela; and the West African species *E. africanus*. The tree is rooted on *G. schimkewitschi*, a member of subfamily Thelyphoninae from Southeast Asia. 

All except one species were fully scored for all characteristics in the matrix (unless females were unavailable). No adult specimens were examined for *R. kovariki*; hence, this species was scored in part from data and illustrations in the original description [55]. The types and only known specimens of *T. ruschii* were not examined. (The holotype male was mislaid or lost, as the last author found a jar containing the specimen label data empty during a visit to the BMNH in 2023.) However, a specimen resembling *T. ruschii* from Guyana, tentatively identified as *T.* aff. *ruschii*, was examined and scored in the matrix. This specimen shares several morphological characteristics in common with *T. ruschii*, including a distinct anteromedian epistome on the carapace (see [48] (p. 112: abb. 3)), but is considerably smaller, resembling the average size of *T. amazonicus*. The Iwokrama Forest Reserve where it was collected is ca. 300 km south of Demerara, the type locality of *T. ruschii*, and separated from it by the Essequibo River.

All characteristics were treated as unordered (i.e., non-additive) and equally weighted. Uninformative characteristics were omitted from the analysis. Tree search was conducted using parsimony with implicit enumeration in TNT, 1.5-beta [60,61] (http://www.lillo.org.ar/phylogeny/tnt/, accessed on 20 May 2024). Nodal support was evaluated with Goodman–Bremer values [62,63], synapomorphies optimized using the accelerated transformation (ACCTRAN) algorithm, and consistency and retention indices calculated in WinClada [64]. Tree files were edited with Figtree 1.4.4 (https://github.com/rambaut/figtree, accessed on 20 May 2024) and Inkscape 1.2.2 (http://www.inkscape.org, accessed on 20 May 2024).

## 3. Results

### 3.1. Phylogenetic Analysis

**Figure 1:**
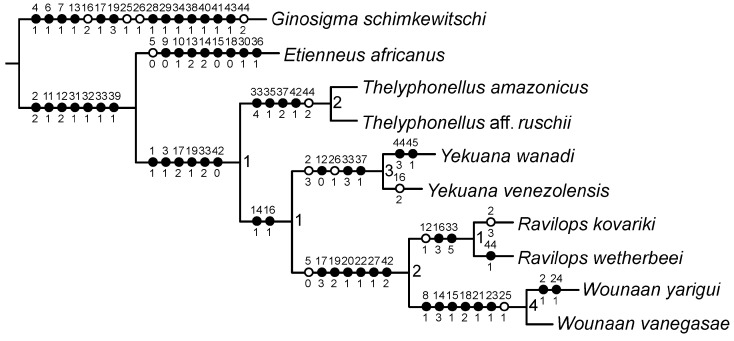
Single most parsimonious tree (L = 53) obtained by analysis of 45 morphological characteristics for ten species of whip-scorpions (Hypoctoninae Pocock, 1899), via implicit enumeration with equal weights. Morphological synapomorphies (using ACCTRAN optimization) illustrated on each branch, with numbers above indicating characteristics and numbers below indicating character states. Solid (black) circles indicate uniquely derived character states, and empty (white) circles indicate parallel derivations. Measure of support (Goodman–Bremer) for branches indicated at nodes. Tree consistency index (CI): 0.87; tree retention index (RI): 0.85. Refer to Appendix B for character matrix and Appendix C for list of characters.

A single most parsimonious tree, 53 steps in length, was obtained by the phylogenetic analysis (Figure 1). The tree topology indicated that *Thelyphonellus* is paraphyletic with respect to *Ravilops*. The New World Hypoctoninae comprises four clades, each with disjunct distributions and well supported by a combination of morphological characteristics, on the basis of which four genera, two of which are new, are recognized: *Ravilops*, with two species, endemic to Hispaniola; *Thelyphonellus*, herein restricted to *T. amazonicus* (Butler, 1872) and *T. ruschii* Weygoldt, 1979, occurring in Guyana, Suriname, French Guiana, and northern Brazil; *Wounaan*, **gen. n.**, containing *Wounaan vanegasae* (Giupponi and Vasconcelos, 2008), **comb. n.** and *Wounaan yarigui*, **sp. n.** from Colombia; and *Yekuana*, **gen. n.**, containing *Yekuana venezolensis* (Haupt, 2009), **comb. n.** and *Yekuana wanadi*, **sp. n.** from Venezuela.

*Thelyphonellus* was supported by four uniquely derived character states (*) and one parallel derivation (^) (characters remain to be confirmed in *T. ruschii*): Char. 33→4*, opisthosomal tergites (♂) I entire, II and III each with distinct median longitudinal suture, complete, IV and to lesser extent V–VIII each with longitudinal suture anteriorly only (obsolete in all but IV), with other tergites undivided. Char. 35→1*, opisthosomal tergites II and III, posterior margin slightly emarginate medially (unlike tergites IV–VIII, linear). Char. 37→2*, opisthosomal sternite II (genital), posterior margin (♂) markedly expanded (enlarged and lobate) and semicircular along entire margin (significantly larger than in female). Char. 42→1*, opisthosomal segment XII (posterior segment of pygidium), dorsolateral ommatoids obsolete, very small and barely visible. Char. 44→2^, opisthosomal flagellum (♂) first segment long (slightly longer than posterior segment of pygidium), with others relatively short.

*Ravilops* was supported by two uniquely derived character states (*) and one parallel derivation (^): Char. 16→3*, pedipalp trochanter, proventral distal tubercle (♂) markedly enlarged (much longer than broad) (confirmed in *R. kovariki*). Char. 33→5*, opisthosomal tergites (♂) I partially divided (posteriorly only), terminating in triangular hyaline area, II and III each with distinct median longitudinal suture, complete, IV and to lesser extent V each with longitudinal suture anteriorly only (obsolete in both), with other tergites undivided (to be confirmed in *R. kovariki*). Char. 12→1^, median sternum (mesosternum) with markedly sclerotized and pigmented area anteriorly only, with the rest of mesosternum pale and depigmented (to be confirmed in *R. kovariki*).

*Yekuana*, **gen. n.** was supported by three uniquely derived character states (*) and two parallel derivations (^): Char. 12→0*, median sternum (mesosternum) markedly sclerotized and pigmented across entirety, entire (not divided longitudinally). Char. 33→3*, opisthosomal tergites (♂) I entire, II and III each with distinct median longitudinal suture, complete, IV and to lesser extent V each with longitudinal suture anteriorly only (obsolete in both), with other tergites undivided. Char. 37→1*, opisthosomal sternite II (genital), posterior margin (♂) moderately expanded (enlarged and lobate) and semicircular along entire margin. Char. 2→3^, carapace anterior margin (♂) markedly pointed. Char. 26→1^, pedipalp movable finger (tarsus), dorsal row of denticles, basal lobe (♂) pronounced.

*Wounaan*, **gen. n.** was supported by six uniquely derived character states (*) and one parallel derivation (^): Char. 8→1*, carapace anteromedian longitudinal raised surface (anterior to median ocular surface, different to superciliary carina) present, moderate (not obscuring anteromedian epistome in dorsal aspect) or pronounced (obscuring anteromedian epistome in dorsal aspect). Char. 14→3*, pedipalp cuticle, microsculpture on dorsal and retrolateral surfaces of segments fundamentally smooth but with fine yet distinct reticulation (visible at great magnification). Char. 15→1*, pedipalp trochanter, principal (fourth) prodorsal tubercle (♂) similar to or shorter than adjacent (third and fifth) tubercles. Char. 18→2*, pedipalp patellar apophysis, length relative to patella width (♂) long, length greater than the patella width. Char. 21→1*, pedipalp tibia (manus) (♂) markedly expanded dorsoventrally (subcircular in lateral aspect, not barrel-shaped). Char. 23→1*, pedipalp tibia (manus), ventral part of retrolateral surface (i.e., retrolateral surface aligned with movable finger), surface (♂) planar or concave. Char. 25→1^, pedipalp fixed (tibial) finger, ventral row of denticles, shape in retrolateral aspect (♂) slightly or markedly sinuous.

The Goodman–Bremer values at nodes equaled 4 for the clade comprising (*W. vanegasae* + *W. yarigui*); 3 for the clade comprising (*Y. venezolensis* + *Y. wanadi*); 2 each for the clades (*Ravilops* + *Wounaan*) and (*T. amazonicus* + *T.* aff. *ruschii*); and 1 each for the clades (*R. kovariki* + *R. wetherbeei*), (*Yekuana* (*Ravilops* + *Wounaan*)), and (*Thelyphonellus* (*Yekuana* (*Ravilops* + *Wounaan*))) (Figure 1).

### 3.2. Systematics

Order Thelyphonida Latreille, 1804

Family Thelyphonidae Lucas, 1835

Subfamily Hypoctoninae Pocock, 1899

#### 3.2.1. Key to the Identification of the Genera of Neotropical Hypoctoninae Pocock, 1899

Characters refer to male and female unless specified otherwise. Refer to the generic diagnoses of *Wounaan* and *Yekuana* for more extensive diagnostic character combinations. Characters yet to be confirmed but presumed to occur in *R. kovariki* and *T. ruschii* (even if confirmed in *T.* aff. *ruschii*) are indicated by (γ) and (δ), respectively. The females of *W. yarigui*, *Y. venezolensis*, and *Y. wanadi* remain unknown.

**Figure 2:**
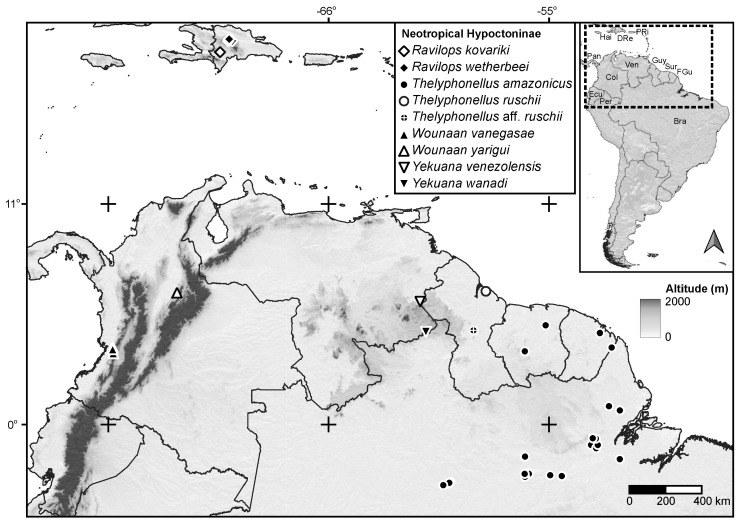
Map of the Caribbean and northern South America, plotting known locality records of Neotropical whip-scorpions (Hypoctoninae Pocock, 1899), based on material examined and published records. Abbreviations: Bra, Brazil; Col, Colombia; DRe, Dominican Republic; Ecu, Ecuador; FGu, French Guiana; Guy, Guyana; Hai, Haiti; Pan, Panama; Per, Peru; PRi, Puerto Rico; Sur, Suriname; Ven, Venezuela.

**Figure 3:**
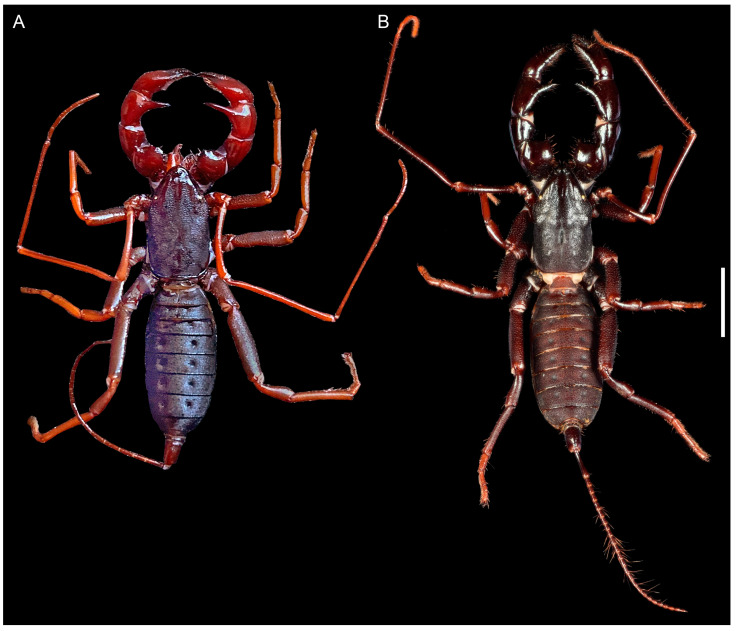
Hypoctoninae Pocock, 1899: habitus, dorsal aspect. (**A**) *Wounaan yarigui*, **gen. et sp. n.**, holotype ♂ (IAvH I 2831). (**B**) *Yekuana wanadi*, **gen. et sp. n.**, holotype ♂ (AMNH IZC 325050). Scale bar = 5 mm.

**Figure 4:**
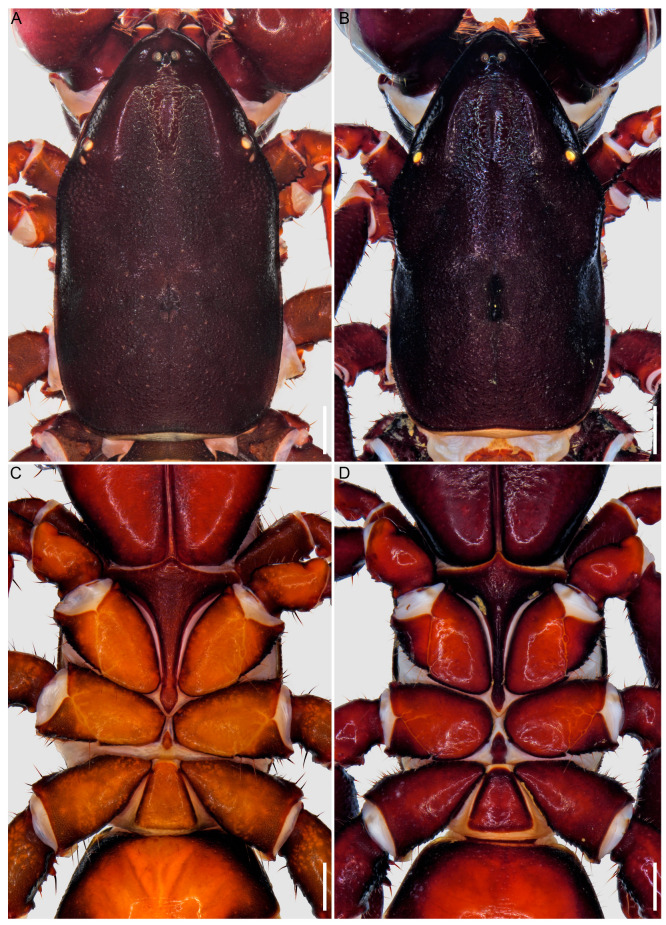
Hypoctoninae Pocock, 1899: carapace, dorsal aspect (**A**,**B**) and coxosternal region, ventral aspect (**C**,**D**). (**A**,**C**) *Wounaan yarigui*, **gen. et sp. n.**, holotype ♂ (IAvH I 2831). (**B**,**D**) *Yekuana wanadi*, **gen. et sp. n.**, holotype ♂ (AMNH IZC 325050). Scale bars = 1 mm.

**Figure 5:**
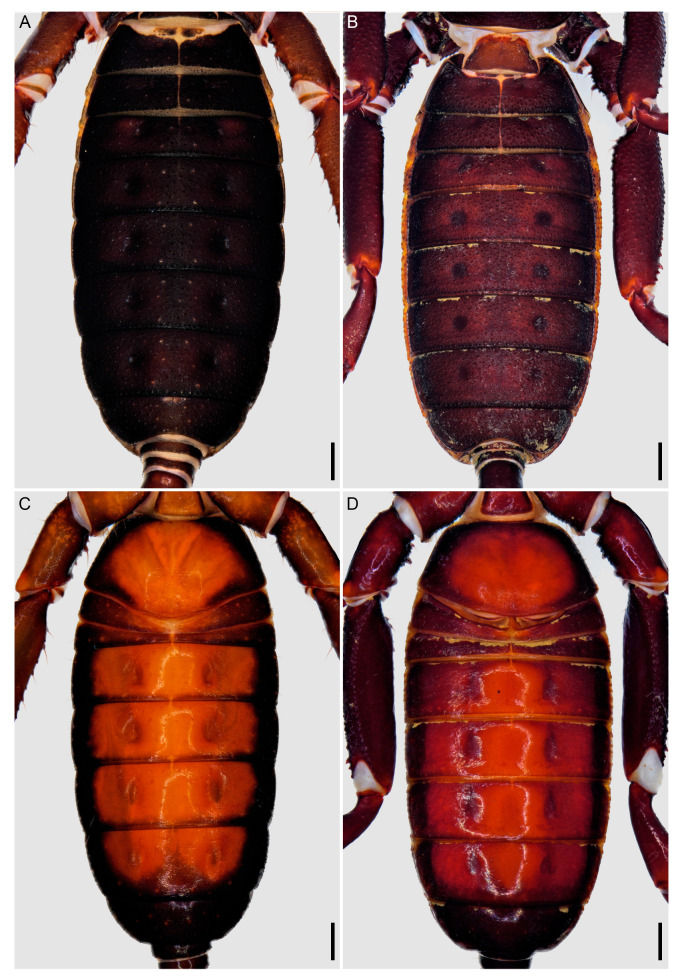
Hypoctoninae Pocock, 1899: opisthosoma, dorsal (**A**,**B**) and ventral (**C**,**D**) aspects. (**A**,**C**) *Wounaan yarigui*, **gen. et sp. n.**, holotype ♂ (IAvH I 2831). (**B**,**D**) *Yekuana wanadi*, **gen. et sp. n.**, holotype ♂ (AMNH IZC 325050). Scale bars = 1 mm.

**Figure 6:**
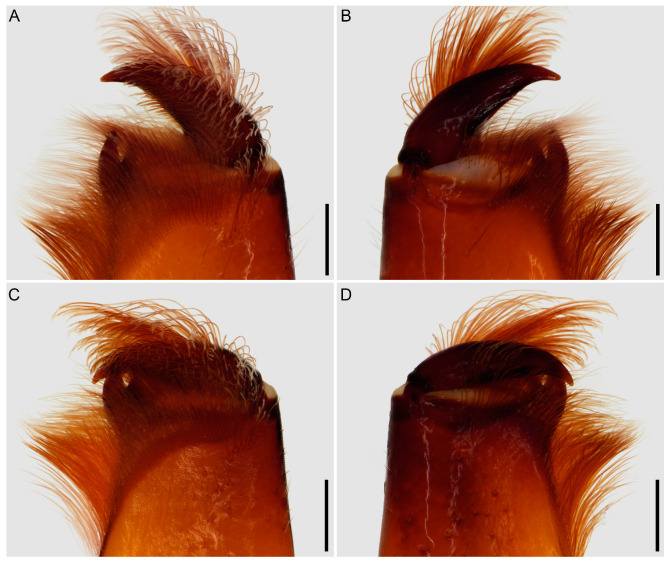
Hypoctoninae Pocock, 1899: chelicera, prolateral (**A**,**C**) and retrolateral (**B**,**D**) aspects. (**A**,**B**) *Wounaan yarigui*, **gen. et sp. n.**, holotype ♂ (IAvH I 2831). (**C**,**D**) *Yekuana wanadi*, **gen. et sp. n.**, holotype ♂ (AMNH IZC 325050). Scale bars = 0.5 mm.

**Figure 7:**
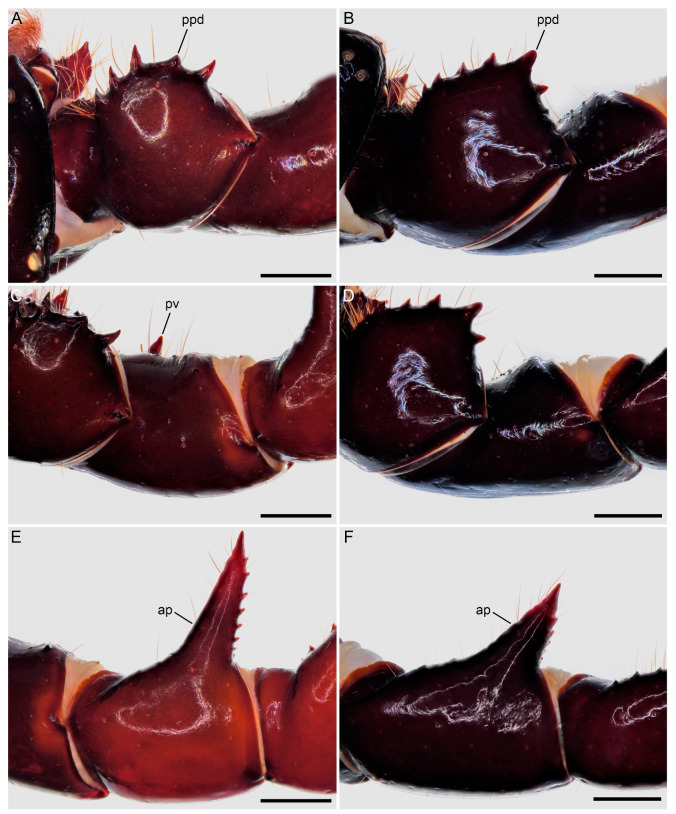
Hypoctoninae Pocock, 1899: pedipalp trochanter (**A**,**B**), femur (**C**,**D**), and patella (**E**,**F**), dorsal aspect. (**A**,**C**,**E**) *Wounaan yarigui*, **gen. et sp. n.**, holotype ♂ (IAvH I 2831). (**B**,**D**,**F**) *Yekuana wanadi*, **gen. et sp. n.**, holotype ♂ (AMNH IZC 325050). Abbreviations: ap, apophysis; ppd, principal prodorsal tubercle; pv, proventral tubercle. Scale bars = 1 mm.

**Figure 8:**
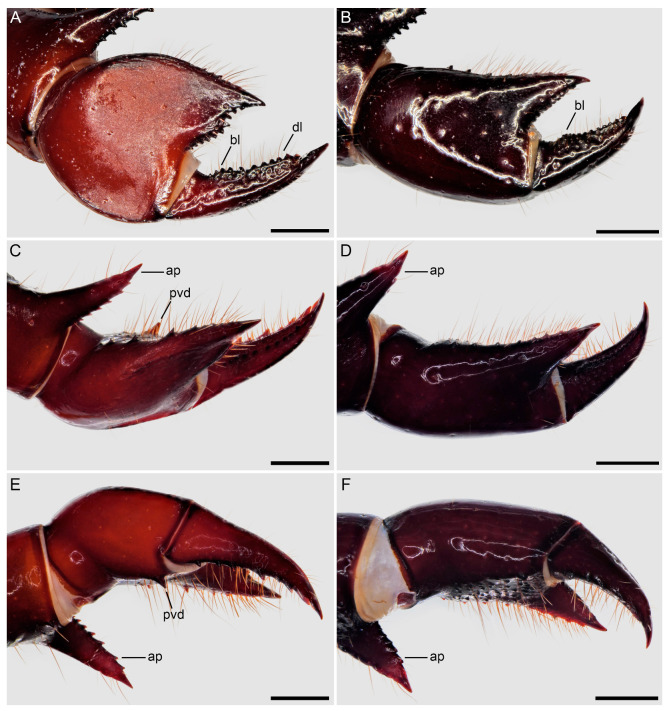
Hypoctoninae Pocock, 1899: pedipalp tibia (manus), retrolateral (**A**,**B**), retrodorsal (**C**,**D**), and proventral (**E**,**F**) aspects. (**A**,**C**,**E**) *Wounaan yarigui*, **gen. et sp. n.**, holotype ♂ (IAvH I 2831). (**B**,**D**,**F**) *Yekuana wanadi*, **gen. et sp. n.**, holotype ♂ (AMNH IZC 325050). Abbreviations: ap, apophysis; bl, basal lobe; dl, distal lobe; pvd, proventral distal tubercle. Scale bars = 1 mm.

**Figure 9:**
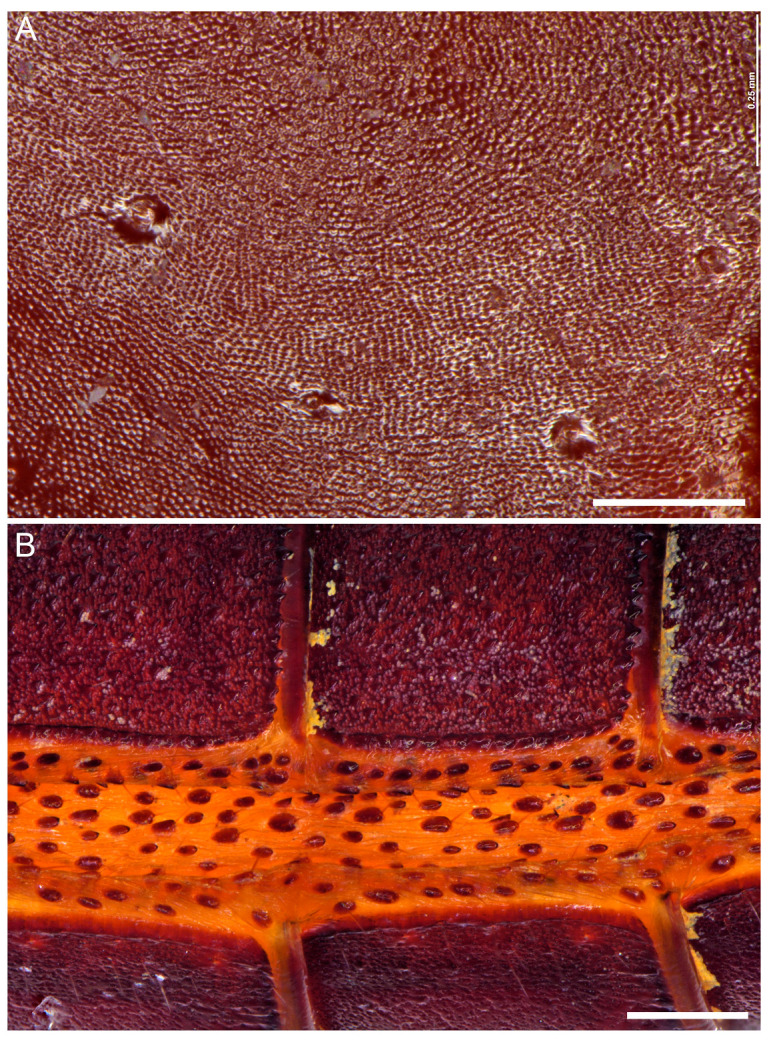
Hypoctoninae Pocock, 1899: pedipalp tibia (manus), retrolateral aspect (**A**) and opisthosoma, lateral aspect (**B**), illustrating surface macro- and microsculpture. (**A**) *Wounaan yarigui*, **gen. et sp. n.**, holotype ♂ (IAvH I 2831). (**B**) *Yekuana wanadi*, **gen. et sp. n.**, holotype ♂ (AMNH IZC 325050). Scale bars = 0.25 mm (**A**), 0.5 mm (**B**).

1. Pedipalp cuticle on dorsal and retrolateral surfaces of segments entirely smooth, without minute reticulation (δ) (Figure 17A); pedipalp trochanter (♂), proventral distal tubercle small, not enlarged (δ) [53] (p. 20: Figure 9); opisthosomal tergites II and III, posterior margin slightly emarginate medially (unlike tergites IV–VIII, which are linear) (δ); opisthosomal sternite II (genital) (♂), posterior margin much expanded (enlarged and lobate) and semicircular along entire margin (significantly larger than in female) (δ) [48] (p. 112: abbs. 6,8), [53] (p. 20: Figure 13) ................................. *Thelyphonellus* Pocock, 1894

– Pedipalp cuticle on dorsal and retrolateral surfaces of segments entirely or predominantly smooth, with fine yet distinct reticulation (visible at great magnification) on segments (*Wounaan*) or chela fingers (*Ravilops*, *Yekuana*) (γ) (Figure 7 and Figure 8; [54] (p. 65: Figure 4), [55] (p. 17: Figure 2)); pedipalp trochanter (♂), proventral distal tubercle moderate (about as long as broad) (*Wounaan*, *Yekuana wanadi*) [53] (p. 19: Figure 2), slightly enlarged (slightly longer than broad) (*Y. venezolensis*) [54] (p. 65: Figures 1 and 2), or markedly enlarged (much longer than broad) (*Ravilops*) [55] (p. 17: Figure 2); opisthosomal tergites II and III, posterior margin unmodified, linear (similar to tergites IV–VIII) (Figure 5A,B; [54] (p. 65: Figure 1), [55] (p. 19: Figure 4A)); opisthosomal sternite II (genital) (♂), posterior margin moderately expanded (enlarged and lobate) (Figure 5C,D and Figure 11A,B; [53] (p. 19: Figure 4), [55] (p. 19: Figure 5A,B)) .......................................................................................................................................................... 2

2. Median sternum (mesosternum) markedly sclerotized and pigmented across entirety (not divided) (Figure 4D and Figure 16F); pedipalp movable finger (tarsus) (♂), dorsal row of denticles with pronounced basal lobe (Figure 8B and Figure 17C); opisthosomal sternite II (genital) (♂), posterior margin moderately expanded (enlarged and lobate) posteriorly, entirely, semicircular (Figure 5D and Figure 11B) ..................................................... *Yekuana*, **gen. n.**

– Median sternum (mesosternum) markedly sclerotized and pigmented anteriorly, rest of mesosternum pale and depigmented (*Ravilops*) (γ) (Figure 16B), or with two markedly sclerotized and pigmented areas, anteriorly and posteriorly, separated by pale, depigmented area medially (posterior pigmented area longitudinally divided or entire) (*Wounaan*) (Figure 16D,E); pedipalp movable finger (tarsus) (♂), dorsal row of denticles with or without obsolete basal lobe (Figure 8A and Figure 17B; [51] (p. 40: Figure 1A)); opisthosomal sternite II (genital) (♂), posterior margin moderately expanded (enlarged and lobate) and sinuous posteromedially (C and A; [53] (p. 19: Figure 4) [55] (p. 19: Figure 5A,B)) ......................................................................................................................... 3

3. Carapace anteromedian surface not raised (γ) [51] (p. 40: Figure 1B), [55] (p. 17: Figure 3B); median sternum (mesosternum) with markedly-sclerotized and pigmented area anteriorly only, rest of mesosternum pale and depigmented (γ) (Figure 16B); pedipalp cuticle, dorsal and retrolateral surfaces of segments entirely smooth [55] (p. 17: Figure 2A,B), except for chela fingers with minute reticulation (visible at great magnification) (γ); pedipalp trochanter (♂), principal (fourth) prodorsal tubercle spiniform, larger than other tubercles, proventral distal tubercle markedly enlarged (much longer than broad) [51] (p. 40: Figure 1A), [55] (p. 17: Figure 2A,B); pedipalp patellar apophysis (♂) slightly shorter than patella width [51] (p. 40: Figure 1A), [55] (p. 17: Figure 2A); pedipalp tibia (manus) (♂) unmodified, not dorsoventrally expanded (barrel-shaped), ventral part of retrolateral surface (i.e., retrolateral surface aligned with movable finger) unmodified, slightly convex like rest of retrolateral surface [51] (p. 40: Figure 1A), [55] (p. 17: Figure 2A,B); pedipalp fixed (tibial) finger (♂), ventral row of denticles linear in retrolateral aspect ........................................................................................... *Ravilops* Víquez and Armas, 2005

– Carapace with anteromedian, moderate or pronounced longitudinal raised surface (anterior to median ocular surface, different to superciliary carina) (Figure 4A); median sternum (mesosternum) with two markedly-sclerotized and pigmented areas, anteriorly and posteriorly, separated by pale depigmented area medially (posterior pigmented area longitudinally divided or entire) (Figure 4C and Figure 16D,E); pedipalp cuticle, dorsal and retrolateral surfaces of segments predominantly smooth, but with fine yet distinct reticulation (visible at great magnification) (Figure 7A,C,E, Figure 8A,C,E, Figure 9A and Figure 17B); pedipalp trochanter (♂), principal (fourth) prodorsal tubercle round, similar to or shorter than adjacent (third and fifth) tubercles (Figure 7A,C; [53] (p. 19: Figures 2 and 3)), proventral distal tubercle moderate (about as long as broad) [53] (p. 19: Figure 2); pedipalp patellar apophysis (♂) length greater than patella width (Figure 7E; [53] (p. 19: Figures 2 and 3)); pedipalp tibia (manus) (♂) markedly expanded dorsoventrally (subcircular in lateral aspect, not barrel-shaped), ventral part of retrolateral surface (i.e., retrolateral surface aligned with movable finger) planar or concave (Figure 8A,C and Figure 17B; [53] (p. 19: Figures 3 and 6)); pedipalp fixed (tibial) finger (♂), ventral row of denticles slightly or markedly sinuous in retrolateral aspect (Figure 8A and Figure 17B) ................................................... *Wounaan*, **gen. n.**

**Figure 10:**
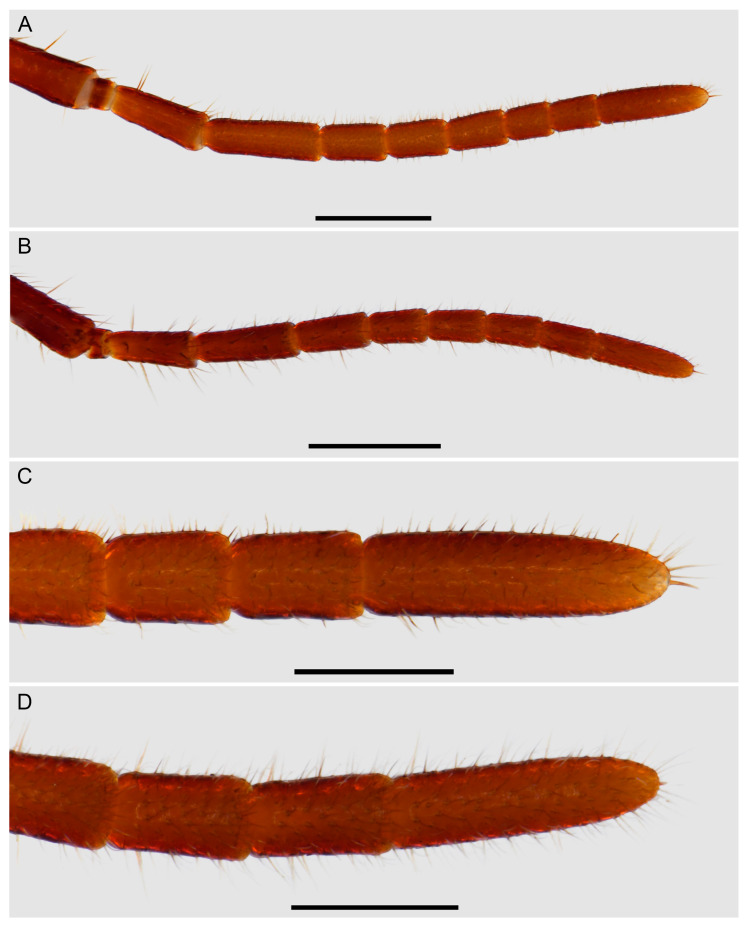
Hypoctoninae Pocock, 1899: leg I tarsus (**A**,**B**) and closeup (**C**,**D**), retroventral (**A**,**C**), prolateral (**B**), and retrodorsal (**D**) aspects. (**A**,**C**) *Wounaan yarigui*, **gen. et sp. n.**, holotype ♂ (IAvH I 2831). (**B**,**D**) *Yekuana wanadi*, **gen. et sp. n.**, holotype ♂ (AMNH IZC 325050). Scale bars = 1 mm (**A**,**B**), 0.5 mm (**C**,**D**).

**Figure 11:**
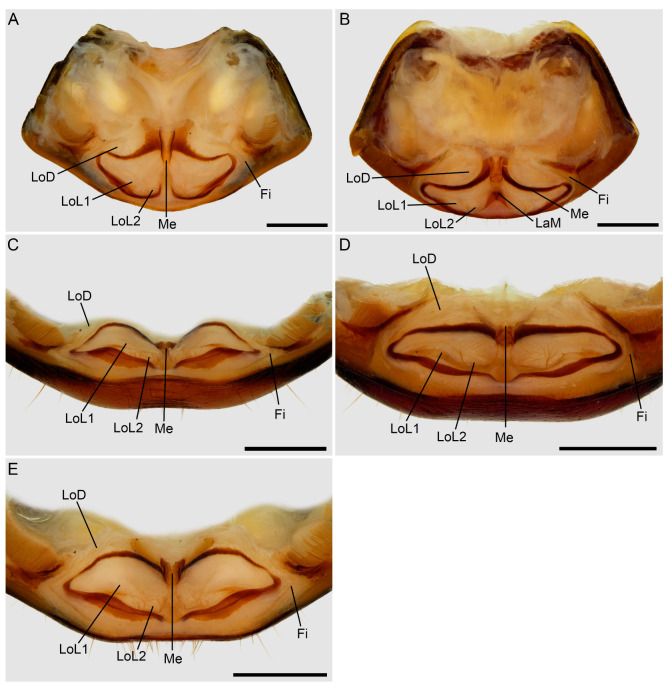
Hypoctoninae Pocock, 1899: dissected sternite II (genital) illustrating internal surface with sclerites and gonopods, dorsal (**A**,**B**), posterodorsal (**C**,**D**), and dorsal, slightly posterior (**E**) aspects. (**A**,**C**,**E**) *Wounaan yarigui*, **gen. et sp. n.**, holotype ♂ (IAvH I 2831). (**B**,**D**) *Yekuana wanadi*, **gen. et sp. n.**, holotype ♂ (AMNH IZC 325050). Abbreviations: Fi, fistula; LaM, lamina medialis; LoD, lobus dorsalis; LoL1, lobus lateralis primus; LoL2, lobus lateralis secundus; Me, mensa. Scale bars = 1 mm.

**Figure 12:**
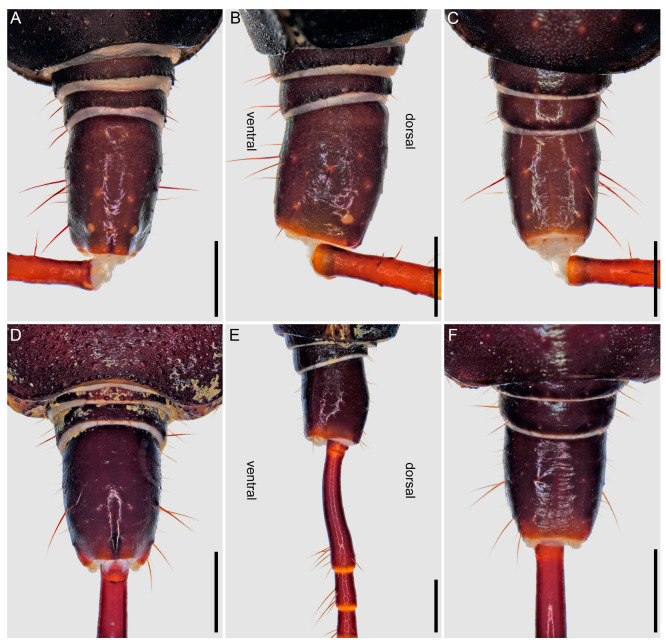
Hypoctoninae Pocock, 1899: opisthosoma, pygidium and base of flagellum (whip), dorsal (**A**,**D**), lateral (**B**,**E**), and ventral (**C**,**F**) aspects. (**A**–**C**) *Wounaan yarigui*, **gen. et sp. n.**, holotype ♂ (IAvH I 2831). (**D**–**F**) *Yekuana wanadi*, **gen. et sp. n.**, holotype ♂ (AMNH IZC 325050). Scale bars = 1 mm.

**Figure 13:**
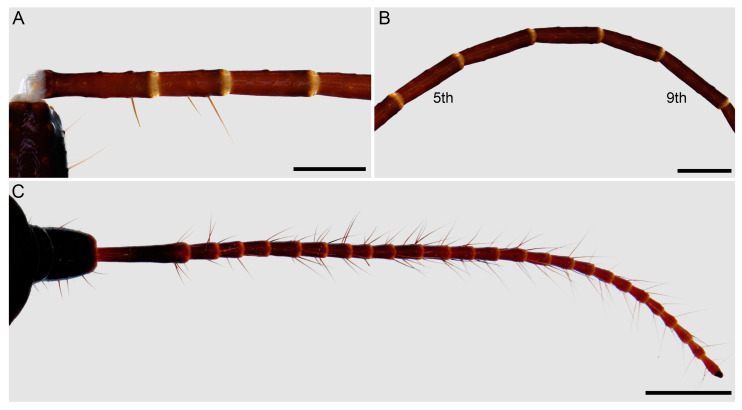
Hypoctoninae Pocock, 1899: opisthosomal flagellum (whip), dorsal (**A**,**B**) and ventral (**C**) aspects. (**A**,**B**) *Wounaan yarigui*, **gen. et sp. n.**, holotype ♂ (IAvH I 2831), proximal segments (**A**) and segments V to IX (**B**). (**C**) *Yekuana wanadi*, **gen. et sp. n.**, holotype ♂ (AMNH IZC 325050), complete flagellum. Scale bars = 1 mm (**A**,**B**), 2 mm (**C**).

**Figure 14:**
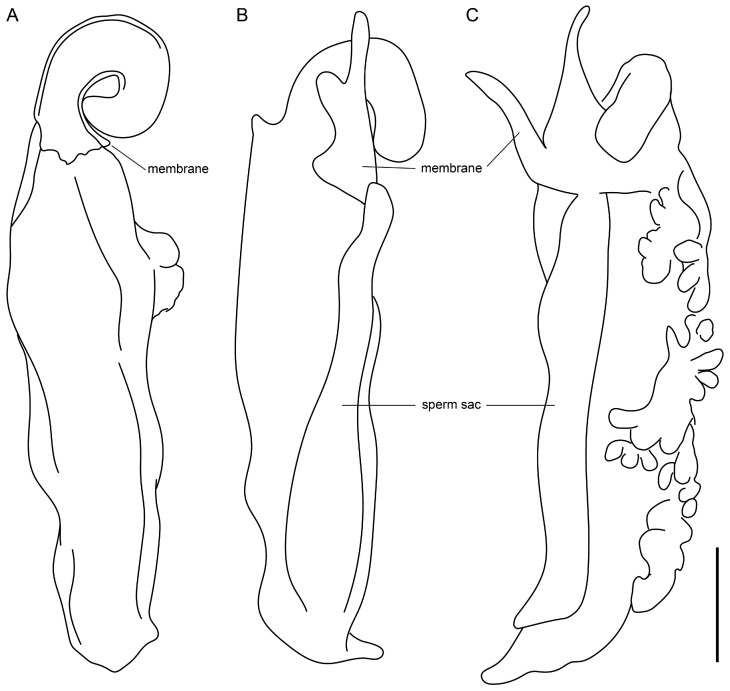
*Yekuana wanadi*, **gen. et sp. n.**, holotype ♂ (AMNH IZC 325050), hemispermatophore, ectal aspect, soft tissues removed (**A**), and ental aspect (**B**,**C**), soft tissues not removed. Scale bar = 1 mm.

**Figure 15:**
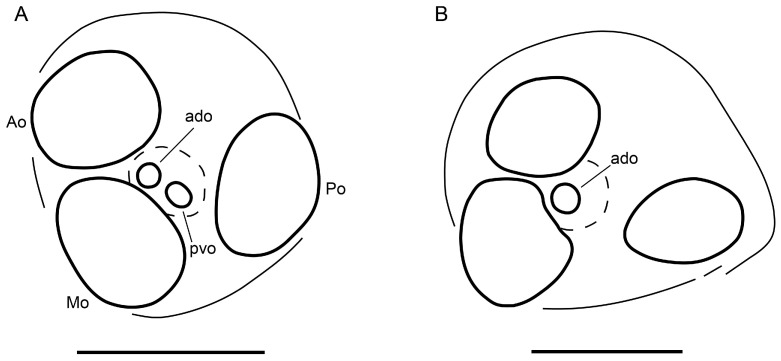
Hypoctoninae Pocock, 1899: lateral (sinistral) ocular tubercle. (**A**) *Thelyphonellus amazonicus* (Butler, 1972), ♂ (MPEG [URO] 9). (**B**) *Etienneus africanus* (Hentschel, 1899), ♀ (AMCC [LP 4654]). Abbreviations: Ao, anterior, Mo, median, and Po, posterior (peripheral) ocelli; ado, anterodorsal, and pvo, posteroventral (central) ocelli. Scale bars = 0.25 mm.

**Figure 16:**
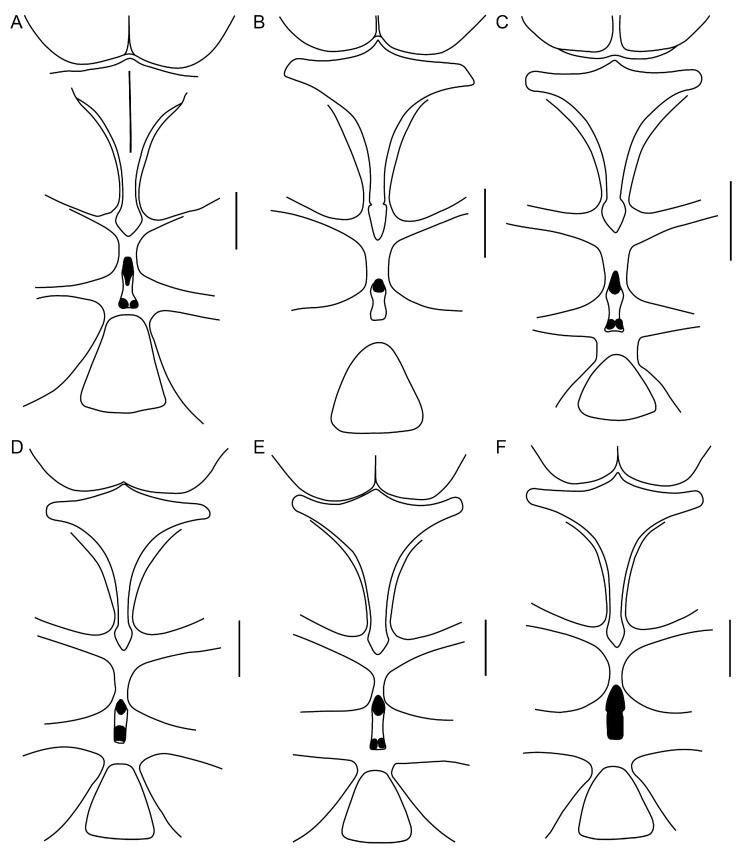
Hypoctoninae Pocock, 1899: coxosternal region, ventral aspect. (**A**) *Etienneus africanus* (Hentschel, 1899), ♀ (AMCC [LP 4654]). (**B**) *Ravilops wetherbeei* (Armas, 2002), ♀ (AMCC [LP 3342]). (**C**) *Thelyphonellus amazonicus* (Butler, 1972), ♀ (MPEG [URO] 7). (**D**) *Wounaan vanegasae* (Giupponi and Vasconcelos, 2008), **comb. n.**, ♂ (MCZ 64455). (**E**) *Wounaan yarigui*, **gen. et sp. n.**, holotype ♂ (IAvH I 2831). (**F**) *Yekuana wanadi*, **gen. et sp. n.**, holotype ♂ (AMNH IZC 325050). Markedly sclerotized parts of the median sternum (mesosternum) indicated in black. Scale bars = 1 mm.

**Figure 17:**
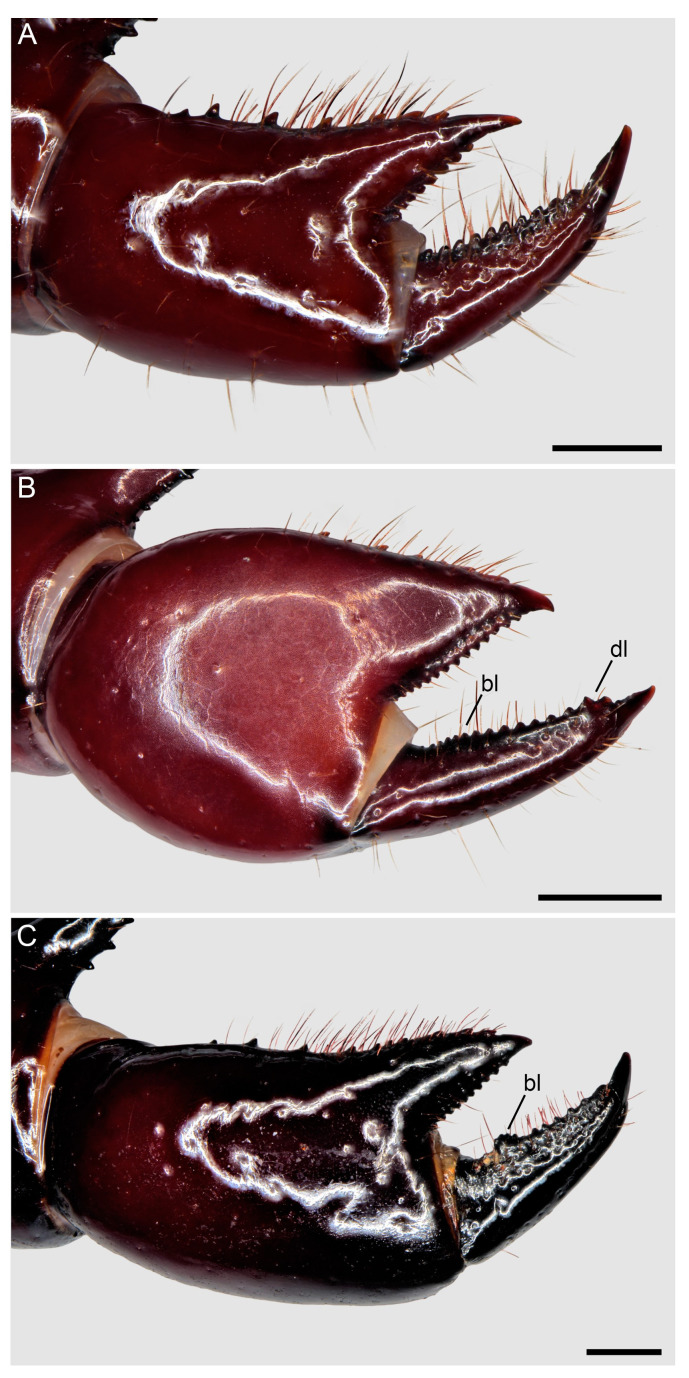
Hypoctoninae Pocock, 1899: pedipalp tibia (manus), retrolateral aspect. (**A**) *Thelyphonellus amazonicus* (Butler, 1972), ♂ (MPEG [URO] 9). (**B**) *Wounaan vanegasae* (Giupponi and Vasconcelos, 2008), **comb. n.**, ♂ (MCZ 64455). (**C**) *Yekuana venezolensis* (Haupt, 2009), **comb. n.**, holotype ♂ (ZMB 48289). Abbreviations: bl, basal lobe; dl, distal lobe. Scale bars = 0.5 mm (**A**), 1 mm (**B**,**C**).

**Figure 18:**
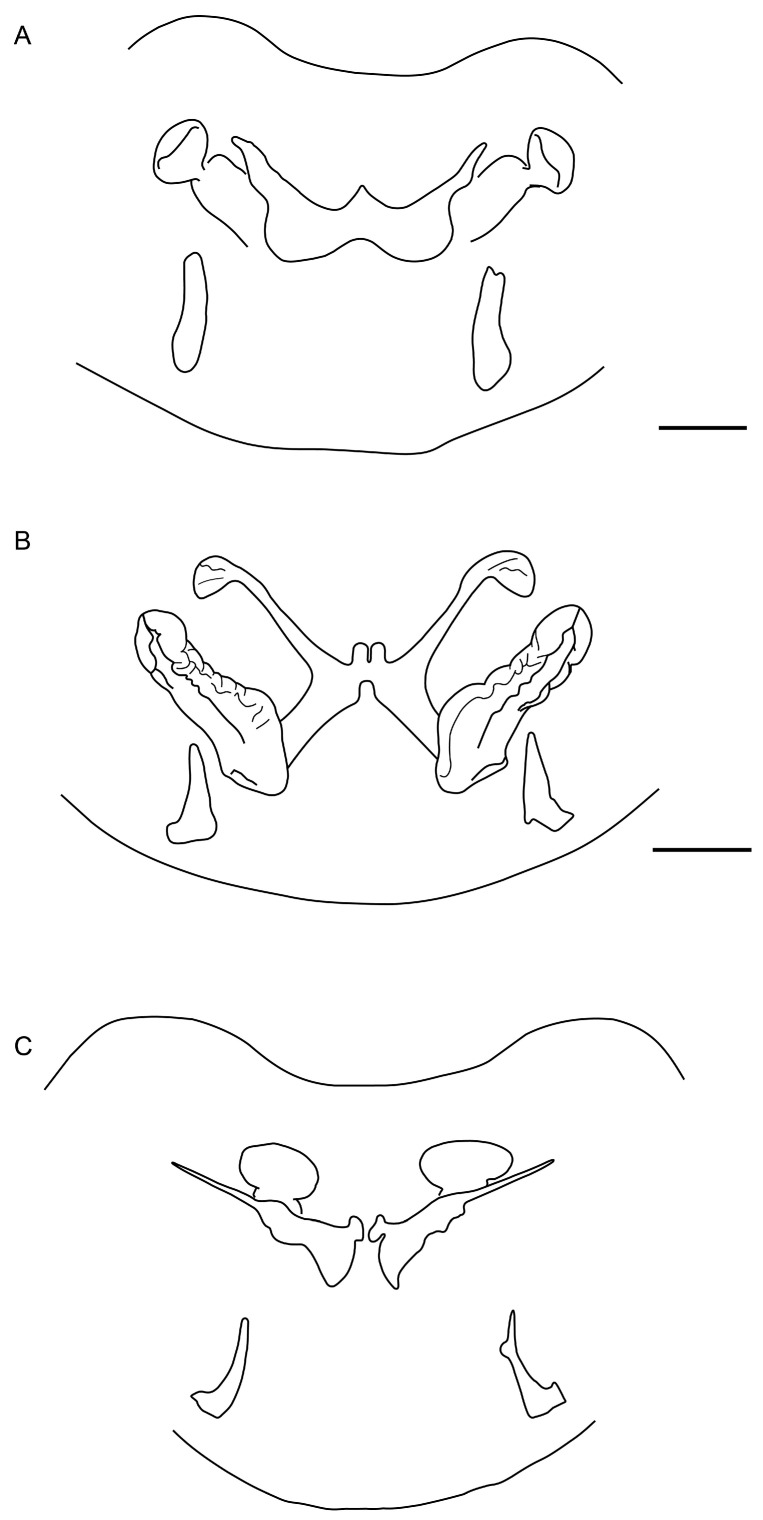
Hypoctoninae Pocock, 1899: dissected sternite II (genital), dorsal aspect, illustrating internal surface with spermathecae. (**A**) *Ravilops wetherbeei* (Armas, 2002), ♀ (AMCC [LP 3342]). (**B**) *Thelyphonellus amazonicus* (Butler, 1972), ♀ (MPEG [URO] 13). (**C**) *Wounaan vanegasae* (Giupponi and Vasconcelos, 2008), **comb. n.**, ♀ (IMCN 9931). Scale bars = 0.5 mm.

**Figure 19:**
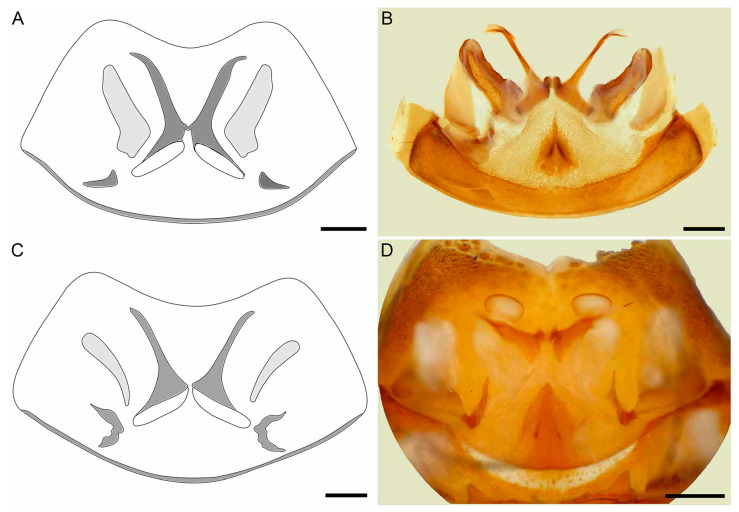
Hypoctoninae Pocock, 1899: dissected sternite II (genital), dorsal aspect, illustrating internal surface with spermathecae. (**A**,**B**) *Thelyphonellus amazonicus* (Butler, 1972), ♀ (SMF 30076), Serra do Navio, Brazil (**A**); ♀ (MPEG [URO] 13), Jari Celulose S.A., Brazil (**B**). (**C**) *Thelyphonellus ruschii* Weygoldt, 1979, paratype ♀ (BMNH, SMF). (**D**) *Wounaan vanegasae* (Giupponi and Vasconcelos, 2008), **comb. n.**, ♀ (IMCN 9931). (**A**,**C**) Redrawn from Weygoldt (1979: 112, abbs. 7,9) [48]. Scale bars = 0.5 mm.

#### 3.2.2. *Wounaan*, **gen. n.**

urn:lsid:zoobank.org:act:4514BBD7-1F35-41EB-BB54-949080F13872

Figure 1, Figure 2, Figure 3A, Figure 4A,C, Figure 5A,C, Figure 6A,B, Figure 7A,C,E, Figure 8A,C,E, Figure 9A, Figure 10A,C, Figure 11A,C,E, Figure 12A–C; Figure 13A,B, Figure 16D,E, Figure 17B, Figure 18C and Figure 19D and Table 1

**Type Species.** *Thelyphonellus vanegasae* Giupponi and Vasconcelos, 2008 [= *Wounaan vanegasae* (Giupponi and Vasconcelos, 2008), **comb. n.**], here designated.

**Diagnosis.** *Wounaan* may be separated from the other Neotropical genera of Hypoctoninae, i.e., *Thelyphonellus*, *Ravilops*, and *Yekuana*, as follows. The anterior margin of the carapace (♂) is slightly pointed or semi-elliptical in *Wounaan* (Figure 4A), whereas it is markedly pointed in *Yekuana* (Figure 4B). The fovea (at least in the ♂) is short, aligned with the trochanter of leg III, and very shallow (barely visible) to moderately shallow (distinct) in *Wounaan* (Figure 4A) but elongated, aligned with the trochanter of leg III, and slightly extending beyond it anteriorly, and deep or moderate in *Thelyphonellus* (at least *T. amazonicus* and *T.* aff. *ruschii*) and *Yekuana* (e.g., Figure 4B). The carapace of *Wounaan* bears a moderate to pronounced, longitudinally raised surface anteromedially (Figure 4A) that is absent in the other genera (e.g., Figure 4B). The median sternum (mesosternum) of *Wounaan* has two markedly sclerotized and pigmented areas, anteriorly and posteriorly, separated by a pale depigmented area medially (Figure 16D,E), whereas the mesosternum of *Yekuana* is markedly sclerotized and pigmented across its entirety (Figure 16F), and that of *Ravilops* (at least *R. wetherbeei*) is only markedly sclerotized and pigmented anteriorly, with the rest of the mesosternum being pale and depigmented (Figure 16B).

**Table 1:** Measurements (mm) for five species of Neotropical Hypoctoninae: *Thelyphonellus amazonicus* (Butler, 1872); *Wounaan vanegasae* (Giupponi and Vasconcelos, 2008), **comb. n.**; *Wounaan yarigui*, **gen. et sp. n.**; *Yekuana venezolensis* (Haupt, 2009), **comb. n.**; and *Yekuana wanadi*, **gen. et sp. n.** Material deposited in the American Museum of Natural History (AMNH), New York, NY, USA; the Instituto de Investigación de Recursos Biológicos “Alexander von Humboldt” (IAvH), Villa de Leyva, Colombia; the Museu Paraense Emílio Goeldi (MPEG), Belém, Brazil; the Museum für Naturkunde der Humboldt-Universität, Berlin (ZMB); and the Museum of Comparative Zoology (MCZ), Harvard University, Cambridge, MA, U.S.A. Abbreviations: L, length; W, width.

		*T. amazonicus*		*W. vanegasae*	*W. yarigui*	*Y. venezolensis*	*Y. wanadi*
Type/Sex		♂	♀	♂	Holotype ♂	Holotype ♂	Holotype ♂
Collection		MPEG [URO] 9	MPEG [URO] 7	MCZ 64455	IAvH I 2831	ZMB 48289	AMNH IZC 325050
Total body L ^1^		17.767	17.44	22.672	20.601	25.833	19.947
Pedipalp ^2^	Trochanter L	2.166	1.913	2.744	2.708	4.513	3.249
	Trochanter W ^3^	1.625	1.444	2.31	2.058	3.357	2.419
	Femur L ^4^	1.083	0.83	1.877	1.625	2.78	1.516
	Femur W ^3^	1.408	1.3	1.877	1.877	2.599	1.877
	Patella L	1.986	1.661	2.527	2.527	4.765	2.96
	Patella W ^5^	1.119	1.011	1.697	1.625	2.527	1.625
	Patellar apophysis L ^6^	0.903	0.758	1.841	1.805	2.022	1.264
	Patellar apophysis W ^7^	0.542	0.505	0.722	0.722	1.155	0.758
	Tibia L ^8^	1.913	1.336	3.791	3.718	6.137	3.61
	Tibia W ^3^	0.866	0.758	1.588	1.444	2.491	1.408
Carapace	L at midline	5.559	5.341	7.834	7.67	10.137	7.562
	W at lateral ocelli	2.611	2.557	3.645	3.59	4.624	3.373
	W at fovea	3.101	2.883	4.461	4.461	5.44	4.08
	Med. oc. tubercle dist ^9^	0.366	0.387	0.645	0.581	0.968	0.624
	Ocular W	0.139	0.139	0.22	0.116	0.197	0.174
	Median ocelli distance	0.186	0.162	0.174	0.197	0.255	0.186
Leg I	Coxa L	1.191	1.264	1.733	1.625	1.986	1.625
	Trochanter L	0.903	0.939	1.372	1.48	1.805	1.336
	Femur L	3.373	3.264	5.451	5.415	6.365	4.549
	Patella L	4.896	4.57	7.834	7.942	9.466	6.8
	Tibia L	5.005	4.733	7.997	7.888	9.302	6.854
	Tarsus L	3.645	3.101	5.005	5.984	5.712	4.57
Leg IV	Coxa L	1.841	1.805	2.563	2.527	3.321	2.419
	Trochanter L	1.913	1.877	2.78	2.635	3.285	2.527
	Femur L	3.502	3.321	5.712	5.277	6.498	4.896
	Patella L	1.913	1.733	2.635	2.527	3.321	2.527
	Tibia L	2.599	2.599	4.765	4.657	4.982	3.971
	Basitarsus L	1.011	1.011	1.372	1.264	1.625	1.336
	Telotarsus L	1.625	1.552	2.31	2.238	2.599	2.166
Genital operculum	L at midline	2.274	1.697	2.563	2.491	2.708	2.455
Pygidial flagellum	1st segment L	1.227	1.227	1.444	1.372	1.769	2.166
	2nd segment L	0.650	0.686	1.300	0.903	1.444	0.650
	3rd segment L	0.650	0.650	1.227	1.119	1.300	0.542

^1^ Measured from anterior carapace margin to posterior edge of pygidium; ^2^ in dorsal aspect; ^3^ maximum width; ^4^ between proximal and distal condyles; ^5^ excluding apophysis; ^6^ measured along dentate (distal) margin; ^7^ at base; ^8^ from base to apex of fixed finger; ^9^ from carapace anterior margin.

Several differences in pedipalp morphology exist between *Wounaan* and the other genera. The cuticle of the pedipalp dorsal and retrolateral surfaces is predominantly smooth but with fine yet distinct reticulation (visible at great magnification) in *Wounaan*, whereas it is entirely smooth in the other genera, except for the chela fingers, which are minutely reticulate (visible at great magnification) in *Ravilops* (at least *R. wetherbeei*) and *Yekuana*. The principal (fourth) prodorsal tubercle of the pedipalp trochanter (♂) is similar to or shorter than the adjacent (third and fifth) tubercles in *Wounaan* (Figure 7A) but larger than the adjacent (third and fifth) tubercles in *Thelyphonellus*, *Ravilops*, and *Yekuana* (e.g., Figure 7B). The proventral distal tubercle of the trochanter (♂) is moderately enlarged (about as long as broad) in *Wounaan*, markedly enlarged (much longer than broad) in *Ravilops*, and small and not enlarged in *Thelyphonellus* (at least *T. amazonicus* and *T.* aff. *ruschii*). The proventral tubercle of the pedipalp femur (♂) is large and spiniform in *Wounaan* (Figure 7C) but moderate and subspiniform in *Thelyphonellus* (at least *T. amazonicus* and *T.* aff. *ruschii*) and *Yekuana* (e.g., Figure 7D). The pedipalp patellar apophysis (♂) is long, its length greater than the patella width, in *Wounaan* (Figure 7E) but moderate, its length slightly less than the patella width, in the other genera (e.g., Figure 7F). The prolateral (anterior) margin of the patellar apophysis (♂) bears a row of 7–9 granules (not including the apex) in *Wounaan* (Figure 7E) compared to a row of 4–5 granules in *Thelyphonellus* and a row of 3–5 granules in *Yekuana* (Figure 7F). The proventral distal tubercle of the patella (♂) is moderate and distinct in *Wounaan*, whereas it is small in *Thelyphonellus* (at least *T. amazonicus* and *T.* aff. *ruschii*) and small or obsolete in *Yekuana*. The pedipalp tibia (manus) (♂) is markedly expanded dorsoventrally (subcircular in lateral aspect, not barrel-shaped) in *Wounaan* (Figure 8A,C and Figure 17B) but unmodified and not dorsoventrally expanded (barrel-shaped) in *Ravilops* (at least *R. wetherbeei*), *Thelyphonellus*, and *Yekuana* (e.g., Figure 8B,D and Figure 17A,C). The proventral distal tubercle of the tibia (manus) (♂) is large and spiniform in *Wounaan* (Figure 8C,E) but small, rounded or subtriangular in *Thelyphonellus* (at least *T. amazonicus* and *T.* aff. *ruschii*) and small and rounded in *Yekuana* (Figure 8F). The ventral part of the retrolateral surface of the tibia (manus) (i.e., the retrolateral surface aligned with the movable finger) (♂) is planar to noticeably concave in *Wounaan* (Figure 8A,C and Figure 17B), whereas it is unmodified and slightly convex, like the rest of the retrolateral surface, in *Ravilops* (at least *R. wetherbeei*), *Thelyphonellus* (at least *T. amazonicus* and *T.* aff. *ruschii*), and *Yekuana* (e.g., Figure 8B,D and Figure 17A,C). The ventral row of denticles on the pedipalp fixed (tibial) finger (♂) is slightly to markedly sinuous in retrolateral aspect in *Wounaan* (Figure 8A and Figure 17B) but linear in retrolateral aspect in *Ravilops*, *Thelyphonellus* (at least *T. amazonicus* and *T.* aff. *ruschii*), and *Yekuana* (Figure 8B and Figure 17A,C). The basal lobe of the dorsal row of denticles on the pedipalp movable finger (tarsus) (♂) is obsolete in *Wounaan* (Figure 8A and Figure 17B) but pronounced in *Yekuana* (Figure 8B and Figure 17C). The dorsal row of denticles on the tarsus (♂) bears a distinct, shallow distal lobe in *Wounaan* (Figure 8A and Figure 17B) that is absent in *Thelyphonellus* (at least *T. amazonicus* and *T.* aff. *ruschii*) and *Yekuana* (Figure 8B and Figure 17A,C).

There are also several differences in the opisthosomal morphology between *Wounaan* and the other genera. Tergite I is entire, II–IV each exhibit a distinct median longitudinal suture (complete in II and III but only present anteriorly in IV), and the other tergites are undivided in *Wounaan* (♂) (Figure 5A), whereas tergite I is entire, II and III each exhibit a distinct median longitudinal suture (complete), IV and to a lesser extent V only exhibit a longitudinal suture anteriorly (obsolete in both), and the other tergites are undivided in *Yekuana* (♂) (Figure 5B); tergite I is entire, II and III each exhibit a distinct median longitudinal suture (complete), IV and to a lesser extent V–VIII only exhibit a longitudinal suture anteriorly (obsolete in all but IV), and the other tergites are undivided in *Thelyphonellus* (at least *T. amazonicus* and *T.* aff. *ruschii*) (♂); and tergite I is partially divided (posteriorly only) and terminating in a triangular hyaline area, II and III each exhibit a distinct median longitudinal suture (complete), IV and to a lesser extent V only exhibit a longitudinal suture anteriorly (obsolete in both), and the other tergites are undivided in *Ravilops* (at least *R. wetherbeei*) (♂). The posterior margin of sternite II (genital) (♂) is moderately expanded (enlarged and lobate) and sinuous posteromedially in *Wounaan* (Figure 5C) but moderately expanded (enlarged and lobate) and semicircular along the entire margin in *Yekuana* (Figure 5D) and markedly expanded (enlarged and lobate) and semicircular along the entire margin (significantly larger than in ♀) in *Thelyphonellus* (at least *T. amazonicus* and *T.* aff. *ruschii*). The opisthosomal segment XII (distal segment of pygidium) of *Wounaan* bears a pair of well-developed, medium-sized dorsolateral ommatoids (Figure 12A,B) that are obsolete, very small and barely visible or absent in *Thelyphonellus* and absent in *Yekuana* (Figure 12D,E).

**Etymology.** The new genus is named in honor of the Wounaan (a.k.a., Wauna, Waunana, Chanco, or Noanamá), a semi-nomadic indigenous tribe inhabiting the Choco biogeographical region of Colombia. The word “Wounaan” in the Embera dialect means “good man, friend-people.” The name is feminine in gender.

**Included Species.** *Wounaan*, **gen. n.** is hereby created to accommodate two species, one of which was formerly assigned to *Thelyphonellus*: *Wounaan vanegasae* (Giupponi and Vasconcelos, 2008), **comb. n.** and *Wounaan yarigui*, **sp. n.**

**Distribution.** Recorded in the Valle del Cauca and Santander departments of Colombia (Figure 2).

##### *Wounaan yarigui*, **sp. n.**

urn:lsid:zoobank.org:act:A482587D-BEBF-4FBE-A34D-BD03ECC944E5

Figure 1, Figure 2, Figure 3A, Figure 4A,C, Figure 5A,C, Figure 6A,B, Figure 7A,C,E, Figure 8A,C,E, Figure 9A, Figure 10A,C, Figure 11A,C,E, Figure 12A–C, Figure 13A,B and Figure 16E and Table 1

**Type Material.** Holotype ♂ (IAvH I 2831), **COLOMBIA:** *Santander Department*: Carmen de Chucurí, Vereda La Belleza, 06°34′13″ N 73°34′15″ W, 844 m, tropical humid forest, pitfall, 22.ii.2018, J.C. Neita, E. Torres, and M.I. Castro.

**Diagnosis.** *Wounaan yarigui* differs from *Wounaan vanegasae* as follows. The anterior margin of the carapace (♂) is semi-elliptical in *W. yarigui* (Figure 4A) but slightly pointed in *W. vanegasae*. The anteromedian raised surface of the carapace (i.e., anterior to the median ocular area) is pronounced, obscuring the anteromedian epistome in dorsal aspect, in *W. yarigui* (at least in the ♂) (Figure 4A) but moderate, not obscuring the epistome, in *W. vanegasae*. The fovea is very shallow and barely visible in *W. yarigui* (at least in the ♂) (Figure 4A) but moderately shallow and distinct in *W. vanegasae*. The posterior pigmented area of the median sternum (mesosternum), which is typically infolded and not exposed, is divided longitudinally in *W. yarigui* (Figure 16E), but entire in *W. vanegasae* (Figure 16D). The pedipalp chela has a conspicuous scabrose surface retrolaterally and, to a lesser extent, dorsally in *W. yarigui* (at least in the ♂) (Figure 8A,C and Figure 9A), whereas the chela is predominantly smooth in *W. vanegasae* (Figure 17B). The ventral part of the retrolateral surface of the pedipalp chela manus (i.e., the retrolateral surface aligned with the movable finger) (♂) is noticeably concave in *W. yarigui* (Figure 8A,C) but planar in *W. vanegasae* (Figure 17B). The retrolateral surface of the pedipalp fixed finger (♂) is planar in *W. yarigui* (Figure 8A,C) but slightly convex, like the retrolateral surface of manus, in *W. vanegasae* (Figure 17B). The ventral row of denticles on the pedipalp fixed finger (♂) is markedly sinuous in retrolateral aspect in *W. yarigui* (Figure 8A) but slightly sinuous in *W. vanegasae* (Figure 17B).

**Etymology.** The specific epithet is a noun in apposition honoring the Yariguí indigenous people, a tribe that once inhabited the cloud forests of the Serranía de los Yariguíes, where the new species was collected.

**Description.** Based on the holotype male (IAvH I 2831). Female unknown.

*Total length*: Adult length, measured from anterior margin of carapace to posterior margin of pygidium (segment XII), 20.6 mm (Figure 3A, Table 1).

*Color*: Carapace and tergites dark reddish brown (Figure 4A and Figure 5C). Sternites yellowish to brown, II–VIII each paler, yellow medially, brown laterally (Figure 5C); IX entirely brown. Pygidium yellowish brown (Figure 12A–C). Flagellum reddish brown, covered with reddish macrosetae, segments with anterior and posterior margins yellow (Figure 13A,B). Pedipalp trochanter, femur, patella, tibia, and tarsus reddish chestnut (Figure 7A,C,E and Figure 8A,C,E); coxae paler (Figure 4C). Legs yellowish to reddish brown, becoming progressively paler distally with tibia and tarsi yellow (Figure 3A and Figure 10A,C).

*Chelicerae*: Movable finger longer than fixed finger, hinged along dorsal margin, prolateral surface with dense brush of long, curved, reddish macrosetae (Figure 6A,B); distal half of manus, prolateral, ventral, and to a lesser extent retrolateral surfaces each with dense brush of sublinear, reddish macrosetae; fixed finger with two well-developed teeth of similar size.

*Prosoma*: Carapace surface scabrose, with shallow granules (Figure 4A); anterior margin semi-elliptical; fovea short, very shallow, aligned with leg III trochanter; anteromedian epistome pronounced, acute; anterior third of carapace with distinct, smooth W-shaped area, without anterolateral oblique carinae between median and lateral ocelli; part of carapace anterior to median ocular surface raised medially, obscuring anteromedian epistome in dorsal aspect (Figure 4A); median ocular area without superciliary carina between ocelli; lateral ocular tubercle with three (anterior, median, and posterior) medium to large, yellow peripheral ocelli surrounding two (anterodorsal and posteroventral) minute, darkened central ocelli (similar to Figure 15A). Anterior sternum (prosternum) without median longitudinal suture (Figure 4C and Figure 16E); posterior stylet-like part relatively broad, arrow-shaped, and completely exposed, not obscured by coxae of legs II. Median sternum (mesosternum) infolded, not completely exposed; markedly sclerotized, pigmented areas anteriorly (exposed) and posteriorly (obscured), separated by pale, depigmented area medially (Figure 16E); posterior pigmented area longitudinally divided.

*Pedipalps*: Surfaces predominantly smooth and shiny, but with fine yet distinct reticulation (visible at great magnification) (Figure 7A,C,E and Figure 8A,C,E). Coxa smooth ventrally; apophysis densely covered with macrosetae, terminating anteriorly in tubercle. Trochanter smooth dorsally and retrolaterally (Figure 7A), coarsely granular prolaterally, and sparsely granular ventrally; prodorsal surface with five tubercles; principal (fourth) tubercle round, shorter than adjacent (third and fifth) tubercles (Figure 7A); proventral surface with two tubercles, proximal tubercle small, distal tubercle moderately enlarged. Femur smooth dorsally (Figure 7C), retrolaterally, and ventrally, predominantly smooth prolaterally; prodorsal surface with or without obsolete tubercle; proventral surface with large spiniform tubercle (Figure 7C). Patella smooth dorsally (Figure 7E), retrolaterally, and ventrally, predominantly smooth prolaterally; proventral surface with moderate distal tubercle, distinct. Patellar apophysis longer than patella width (Figure 7E and Table 1); anterior margin with serrate row of 7 or 8 granules; posterior margin without granules. Tibia bulky (not barrel-shaped), manus markedly expanded dorsoventrally, subcircular in lateral aspect (Figure 8A,C,E); retrolateral surface and to a lesser extent dorsal surface with conspicuous scabrose surface covering large part of manus and extending onto fixed finger (Figure 8A,C and Figure 9A), prolateral surface sparsely granular, ventral surface smooth; manus prodorsal margin with row of prominent granules extending onto fixed finger (Figure 8C); proventral surface with large, spiniform distal tubercle (Figure 8C,E); ventral part of retrolateral surface of manus (i.e., retrolateral surface aligned with movable finger) noticeably concave (Figure 8A,C). Fixed finger, ventral margin with serrate row of denticles; retrolateral surface planar (Figure 8A,C); row of denticles markedly sinuous in retrolateral aspect (Figure 8A). Tarsus (movable finger), dorsal margin with serrate row of denticles and obsolete basal lobe (Figure 8A); distal lobe shallow (possibly produced by subtle median emargination of denticle row); proventral margin with serrate row of denticles progressively increasing in size distally (Figure 8C,E); prolateral surface with smooth longitudinal carina between denticle rows.

*Legs*: Leg I tarsus, first tarsomere shortest, shorter than wide, fourth to sixth about as long as wide, seventh and eighth slightly longer than wide, and second, third, and ninth about three times longer than wide (Figure 10A,C); ninth tarsomere terminating in single claw (or clawlike seta) (Figure 10C). Legs II–IV basitarsi each with proventral and retroventral spurs distally; telotarsi each with ventral macrosetae setiform and arranged irregularly, not in rows. Leg IV tibia with proventral spur distally. Tibia dorsal surface with one (legs II–IV) or two (leg I) trichobothria distally.

*Opisthosoma*: Tergites surface scabrose, densely granular (Figure 5A); I undivided (entire), II–IV each with distinct median longitudinal suture, complete (II and III) or partial, anteriorly only (IV), V–IX undivided (entire); II and III unmodified, each similar in length to IV, or II slightly longer; II–VIII, posterior margins unmodified, linear (not emarginate). Pleural membranes densely covered with markedly sclerotized, elongated granules (similar to Figure 9B). Sternites densely punctate, lateral margins scabrose (Figure 5C); II (genital) undivided (entire), dorsal (internal) surface with genital sclerites relatively simple (Figure 11A,C,E), posterior margin moderately enlarged and lobate (dilate), especially medially, border sinuous (Figure 5C); III and IV each with median longitudinal suture, weakly defined on III, complete on IV, otherwise unmodified; V–VIII each with median longitudinal suture vestigial (anteriorly only); IX undivided (entire); V–IX unmodified. Segments X–XII forming narrow, annular pygidium (Figure 12A–C); XII (anal segment), dorsal surface slightly angular posteriorly, dorsolateral ommatoids well developed, medium-sized, circular (Figure 12A,B). Pygidial flagellum comprising at least 13 segments (additional segments may be missing); each segment with numerous macrosetae (some missing), without ventromedian ommatoids; segment length usually three to five times the maximum width but up to eight or ten times the maximum width in some cases (Figure 13A,B); similar to or shorter than posterior segment of pygidium (XII); basal (first) segment unmodified, linear in lateral aspect.

*Male gonopods*: Chitinized arches and gonopods as in Figure 11A,C,E. LoD circular, flat, and membranous; Fi medium, subtriangular, with sclerotized lateral tips; LoL1 1.5 times broader than long, trapezoidal, membranous, globose, with vestigial sclerotized wrinkles and rounded posterior margin not extending beyond posterior margin of chitinized arch; LoL2 membranose, flat, covered by LoL1; Me 2.2 times longer than wide, subcylindrical, sclerotized laterally and becoming thinner posteriorly; LaM, Fu, and Pi absent. Chitinized arches of LoD and LoL1/LoL2 fused; arches separate, not fused anteromedially and posteromedially; chitinized arch of LoD broad anteromedially; anterior and lateral margins of chitinized arch of LoL1/Lol2 thin, posterior margin thicker.

**Distribution.** *Wounaan yarigui* is known only from the type locality, Carmen de Chucurí, in the Santander Department of Colombia (Figure 2).

#### 3.2.3. *Yekuana*, **gen. n.**

urn:lsid:zoobank.org:act:68003E41-7E5F-4829-8884-B88080CCAE31

Figure 1, Figure 2, Figure 3B, Figure 4B,D, Figure 5B,D, Figure 6C,D, Figure 7B,D,F, Figure 8B,D,F, Figure 9B, Figure 10B,D, Figure 11B,D, Figure 12D–F, Figure 13C, Figure 14, Figure 16F and Figure 17C and Table 1

**Type Species.** *Yekuana wanadi*, **sp. n.**, here designated.

**Diagnosis.** *Yekuana* may be separated from the other Neotropical genera of Hypoctoninae, i.e., *Wounaan*, *Thelyphonellus*, and *Ravilops*, as follows. The anterior margin of the carapace (♂) is markedly pointed in *Yekuana* (Figure 4B), whereas it is slightly pointed in *Thelyphonellus* (at least *T. amazonicus* and *T.* aff. *ruschii*) and slightly pointed or semi-elliptical in *Wounaan* (Figure 4A). The fovea (at least in the ♂) is elongated, aligned with the trochanter of leg III and slightly extending beyond it anteriorly, and deep in *Yekuana* (Figure 4B) but short, aligned with the trochanter of leg III, and very shallow (barely visible) to moderately shallow (distinct) in *Ravilops* (at least *R. wetherbeei*) and *Wounaan* (e.g., Figure 4A). The carapace of *Yekuana* does not possess a longitudinal raised surface anteromedially (Figure 4B), as in *Wounaan* (Figure 4A). The median sternum (mesosternum) of *Yekuana* is markedly sclerotized and pigmented across its entirety (Figure 4D and Figure 16F), whereas the mesosternum of *Thelyphonellus* (at least *T. amazonicus* and *T.* aff. *ruschii*) (Figure 16C) and *Wounaan* (Figure 16D,E) has two markedly-sclerotized and pigmented areas, anteriorly and posteriorly, separated by a pale depigmented area medially, and that of *Ravilops* (at least *R. wetherbeei*) (Figure 16B) is only markedly sclerotized and pigmented anteriorly, with the rest of the mesosternum being pale and depigmented.

Several differences in pedipalp morphology exist between *Yekuana* and the other genera. The cuticle of the pedipalp dorsal and retrolateral surfaces is entirely smooth, except for the chela fingers, which are minutely reticulate (visible at great magnification) in *Yekuana*, whereas it is entirely smooth in *Thelyphonellus* (at least *T. amazonicus* and *T.* aff. *ruschii*) and predominantly smooth but with fine yet distinct reticulation (visible at great magnification) in *Wounaan*. The principal (fourth) prodorsal tubercle of the pedipalp trochanter (♂) is larger than the other tubercles in *Yekuana* (Figure 7B), whereas the tubercle is similar to or shorter than the adjacent (third and fifth) tubercles in *Wounaan* (Figure 7A). The proventral distal tubercle of the trochanter (♂) is moderately enlarged (about as broad as long) or slightly enlarged (slightly longer than broad) in *Yekuana* but markedly enlarged (much longer than broad) in *Ravilops* and small and not enlarged in *Thelyphonellus* (at least *T. amazonicus* and *T.* aff. *ruschii*). The proventral tubercle of the pedipalp femur (♂) is moderate and subspiniform in *Yekuana* but large and spiniform in *Ravilops* (at least *R. wetherbeei*) and *Wounaan* (Figure 7C). The pedipalp patellar apophysis (♂) is moderate, its length slightly less than the patella width, in *Yekuana* (Figure 7F) but long, its length greater than the patella width, in *Wounaan* (Figure 7E). The prolateral (anterior) margin of the patellar apophysis (♂) bears a row of 3–5 granules (not including the apex) in *Yekuana* (Figure 7F) compared to a row of 6–9 granules in *Ravilops* and 7–9 granules in *Wounaan* (Figure 7E). The patella proventral distal tubercle (♂) is small or obsolete in *Yekuana* (Figure 8F), whereas it is moderate and distinct in *Ravilops* (at least *R. wetherbeei*) and *Wounaan* (e.g., Figure 8E). The pedipalp tibia (manus) (♂) is unmodified and not dorsoventrally expanded (barrel-shaped) in *Yekuana* (Figure 8B,D and Figure 17C) but markedly expanded dorsoventrally (subcircular in lateral aspect, not barrel-shaped) in *Wounaan* (Figure 8A,C and Figure 17B). The proventral distal tubercle of the pedipalp tibia (manus) (♂) is small and rounded in *Yekuana* (Figure 8F) but large and spiniform in *Ravilops* and *Wounaan* (e.g., Figure 8C,E). The ventral part of the retrolateral surface of the tibia (manus) (i.e., the retrolateral surface aligned with the movable finger) (♂) is unmodified and slightly convex, like the rest of the retrolateral surface, in *Yekuana* (Figure 8B,D and Figure 17C), whereas it is planar to noticeably concave in *Wounaan* (Figure 8A,C and Figure 17B). The ventral row of denticles on the pedipalp fixed (tibial) finger (♂) is linear in retrolateral aspect in *Yekuana* (Figure 8B and Figure 17C) but slightly to markedly sinuous in retrolateral aspect in *Wounaan* (Figure 8A and Figure 17B). The basal lobe of the dorsal row of denticles on the pedipalp movable finger (tarsus) (♂) is pronounced in *Yekuana* (Figure 8B and Figure 17C) but obsolete or absent in *Ravilops*, *Thelyphonellus* (at least *T. amazonicus* and *T.* aff. *ruschii*), and *Wounaan* (e.g., Figure 8A and Figure 17A,B). The dorsal row of denticles on the tarsus (♂) lacks a distal lobe in *Yekuana* (Figure 8B and Figure 17C) that is present (though small or shallow) in *Ravilops* (at least *R. wetherbeei*) and *Wounaan* (e.g., Figure 8A and Figure 17B).

There are also several differences in the opisthosomal morphology between *Yekuana* and the other genera. Tergite I is entire, II and III each exhibit a distinct median longitudinal suture (complete), IV and to a lesser extent V only exhibit a longitudinal suture anteriorly (obsolete in both), and the other tergites are undivided in *Yekuana* (♂) (Figure 5B), whereas tergite I is partially divided (posteriorly only) and terminating in a triangular hyaline area, II and III each exhibit a distinct median longitudinal suture (complete), IV and to a lesser extent V only exhibit a longitudinal suture anteriorly (obsolete in both), and the other tergites are undivided in *Ravilops* (at least *R. wetherbeei*) (♂); tergite I is entire, II and III each exhibit a distinct median longitudinal suture (complete), IV and to lesser extent V–VIII only exhibit a longitudinal suture anteriorly (obsolete in all but IV), and the other tergites are undivided in *Thelyphonellus* (at least *T. amazonicus* and *T.* aff. *ruschii*) (♂); and tergite I is entire, II–IV each exhibit a distinct median longitudinal suture (complete in II and III and only present anteriorly in IV), and the other tergites are undivided in *Wounaan* (♂) (Figure 5A). The posterior margin of sternite II (genital) (♂) is moderately expanded (enlarged and lobate) and semicircular along the entire margin in *Yekuana* (Figure 5D and Figure 11B) but moderately expanded (enlarged and lobate) and sinuous posteromedially in *Wounaan* and *Ravilops* (e.g., Figure 5C and Figure 11A) and markedly expanded (enlarged and lobate) and semicircular along the entire margin (significantly larger than in ♀) in *Thelyphonellus* (at least *T. amazonicus* and *T.* aff. *ruschii*). The opisthosomal segment XII (distal segment of pygidium) of *Yekuana* lacks dorsolateral ommatoids (Figure 12D,E), unlike *Ravilops* and *Wounaan*, in which a pair of well-developed, medium-sized ommatoids is present (e.g., Figure 12A,B).

**Etymology.** The new genus is named in honor of the Ye’kuana, an indigenous tribe inhabiting the tropical forests of the Orinoco Basin in southern Venezuela (Bolívar State) and a small part of northern Brazil. The name is feminine in gender.

**Included Species.** *Yekuana*, **gen. n.** is hereby created to accommodate two species, one of which was formerly assigned to *Thelyponellus*: *Yekuana venezolensis* (Haupt, 2009), **comb. n.** and *Yekuana wanadi*, **sp. n.**

An unidentified, adult male of *Yekuana* from Sifontes, a municipality in the Bolívar State of Venezuela, was examined during the study. The specimen is significantly smaller than the holotype of *Y. venezolensis* and resembles the holotype of *Y. wanadi* in several respects, including its smaller size (although slightly larger than the holotype of *Y. wanadi*) and similar shape of the anterior margin of the carapace and development of the pedipalp tubercles. Unfortunately, the specimen lacks the flagellum (apparently severed when the specimen was alive based on the presence of a scar), which could have enabled its identification to species, given the marked differences in flagellar morphology between the two species of *Yekuana*. The locality at which the unidentified specimen was collected is near the type locality of *Y. venezolensis* and far from that of *Y. wanadi*.

**Distribution.** Known only from the state of Bolívar, Venezuela (Figure 2).

##### *Yekuana wanadi*, **sp. n.**

urn:lsid:zoobank.org:act:5E94C048-6BB1-45AF-B8B7-2A23CA67296C

Figure 1, Figure 2, Figure 3B, Figure 4B,D, Figure 5B,D, Figure 6C,D, Figure 7B,D,F, Figure 8B,D,F, Figure 9B, Figure 10B,D, Figure 11B,D, Figure 12D–F, Figure 13C, Figure 14 and Figure 16F and Table 1

**Type Material.** Holotype ♂ (AMNH IZC 325050), **VENEZUELA:** *Edo. Bolívar*: St. Elena de Uairén [Santa Elena de Uairén, 04°36′23.2″ N 61°06′19.4″ W], km 315, 14.xi.2005–ii.2006, C. Seiderman.

**Diagnosis.** *Yekuana wanadi* differs from *Y. venezolensis* as follows. *Yekuana wanadi* is considerably smaller than *Y. venezolensis* (at least the ♂) (Table 1). The anterior margin of the carapace (♂) of *Y. wanadi* is less markedly pointed (Figure 4B) than that of *Y. venezolensis*. The proventral distal tubercle on the pedipalp trochanter (♂) is moderate, about as long as broad in *Y. wanadi* but slightly enlarged and longer than broad in *Y. venezolensis*. The anterior margin of the pedipalp patellar apophysis (♂) is armed with five granules in *Y. wanadi* (Figure 7F) but with three or four in *Y. venezolensis*. The first segment of the pygidial flagellum (♂) is very long, noticeably longer than the posterior segment of the pygidium (XII), and the other segments are short, about one-quarter the length of the first flagellar segment or less, in *Y. wanadi* (Figure 12E and Figure 13C), whereas all segments of the flagellum are moderately elongated, similar to or shorter than the posterior segment of the pygidium, in *Y. venezolensis*. The first segment of the pygidial flagellum (♂) is sinuous and broadens posteriorly in lateral aspect in *Y. wanadi* (Figure 12E) but linear and unmodified in lateral aspect in *T. venezolensis*.

**Etymology.** The specific epithet is a noun in apposition inspired by the Venezuelan myth of the “Wanadi”, the Creator, which tells a story of the Wanadi’s wish to make good people on Earth.

**Description.** Based on the holotype male (AMNH IZC 325050). Female unknown.

*Total length*: Adult length, measured from anterior margin of carapace to posterior margin of pygidium (segment XII), 19.95 mm (Figure 3B, Table 1).

*Color*: Carapace dark brown to blackish (Figure 4B); tergites dark reddish brown (Figure 5B). Sternites dark red, II–VIII each with medial part paler, orange-brown (Figure 5D). Pygidium dark reddish brown (Figure 12D–F). Flagellum reddish brown, covered with reddish macrosetae, segments with basal and distal margins yellow (Figure 12E and Figure 13C). Pedipalp trochanter, femur, patella, tibia, and tarsus dark reddish brown (Figure 7B,D,F and Figure 8B,D,F); coxae paler, reddish brown (Figure 4D). Legs dark red, becoming progressively paler distally with tarsi yellowish (Figure 3B and Figure 10B,D).

*Chelicerae*: Movable finger longer than fixed finger, hinged along dorsal margin, prolateral surface with dense brush of long, curved, reddish macrosetae (Figure 6C,D); distal half of manus, prolateral, ventral, and to a lesser extent retrolateral surfaces each with dense brush of sublinear reddish macrosetae; fixed finger with two well-developed teeth of similar size.

*Prosoma*: Carapace surface scabrose, punctate with shallow granules (Figure 4B); anterior margin markedly pointed; fovea elongated, deep, aligned with leg III trochanter and extending slightly anteriorly; anteromedian epistome pronounced, acute; anterior third of carapace with distinct, smooth W-shaped area, without anterolateral oblique carinae between median and lateral ocelli; part of carapace anterior to median ocular surface not raised anteromedially (Figure 4B); median ocular area without superciliary carina between ocelli; lateral ocular tubercle with three (anterior, median, and posterior) medium to large, yellow peripheral ocelli surrounding two (anterodorsal and posteroventral) minute, darkened central ocelli (similar to Figure 15A). Anterior sternum (prosternum) without median longitudinal suture (Figure 44D and Figure 16F); posterior stylet-like part relatively broad, arrow-shaped, and completely exposed, not obscured by coxae of legs II. Median sternum (mesosternum) infolded, not completely exposed (posterior part obscured), markedly sclerotized and entirely pigmented (Figure 16F).

*Pedipalps*: Surfaces predominantly smooth and shiny (Figure 7B,D,F and Figure 8B,D,F). Coxa smooth ventrally; apophysis densely covered with macrosetae, terminating anteriorly in tubercle. Trochanter smooth dorsally and retrolaterally (Figure 7B), coarsely granular prolaterally, and sparsely granular ventrally; prodorsal surface with five tubercles plus small supernumerary tubercle basally on dextral pedipalp; principal (fourth) tubercle largest, spiniform (Figure 7B); proventral surface with two tubercles, proximal tubercle small, distal tubercle moderately enlarged. Femur smooth dorsally (Figure 7D), retrolaterally, and ventrally, coarsely granular prolaterally; prodorsal surface with obsolete tubercle; proventral surface with moderate, subspiniform tubercle. Patella smooth dorsally (Figure 7F), retrolaterally, and ventrally, coarsely granular prolaterally; proventral surface with small distal tubercle. Patellar apophysis slightly shorter than patella width (Figure 7F, Table 1); anterior margin with serrate row of five granules; posterior margin without granules. Tibia unmodified (Figure 8B,D,F); smooth dorsally, retrolaterally, and ventrally, coarsely granular prolaterally; manus barrel-shaped, prodorsal margin with row of prominent granules extending onto fixed finger (Figure 8D); proventral surface with small, rounded distal tubercle (Figure 8F); retrolateral surface unmodified, convex (Figure 8B,D). Fixed finger, ventral margin with serrate row of denticles; retrolateral surface unmodified, slightly convex like retrolateral surface of manus (Figure 8B,D); row of denticles linear in retrolateral aspect (Figure 8B). Tarsus (movable finger), dorsal margin with serrate row of denticles and pronounced basal lobe (Figure 8B); distal lobe absent; proventral margin with serrate row of denticles progressively increasing in size distally; prolateral surface with smooth longitudinal carina between denticle rows.

*Legs*: Leg I tarsus, first tarsomere shortest, shorter than wide, fifth to eighth slightly longer than wide, fourth more than twice as long as wide, and second, third, and ninth about three times longer than wide (Figure 10B,D); ninth tarsomere terminating in single claw (or clawlike seta) (Figure 10D). Legs II–IV basitarsi each with proventral and retroventral spurs distally; telotarsi each with ventral macrosetae setiform and arranged irregularly, not in rows. Leg IV tibia with proventral spur distally. Tibia dorsal surface with one (legs II–IV) or two (leg I) trichobothria distally.

*Opisthosoma*: Tergites surface scabrose, densely granular (Figure 5B); I undivided (entire), II and III each with distinct median longitudinal suture, complete, IV and to a lesser extent V each with median longitudinal suture anteriorly only (obsolete in both), VI–IX undivided (entire); II and III unmodified, each similar in length to IV, or II slightly longer; II–VIII, posterior margins unmodified, linear (not emarginate). Pleural membranes with abundant, markedly sclerotized, elongated granules (Figure 9B). Sternites densely punctate (Figure 5D); II (genital) undivided (entire), dorsal (internal) surface with genital sclerites relatively simple (Figure 11B,D), posterior margin moderately enlarged and lobate (dilate), semicircular (Figure 5D); III and IV each with median longitudinal suture nearly complete (III) or complete (IV), otherwise unmodified; V–VIII each with median longitudinal suture vestigial (anteriorly only); IX undivided (entire); V–IX unmodified. Segments X–XII forming narrow, annular pygidium (Figure 12D–F); XII (anal segment), dorsal surface slightly angular posteriorly, dorsolateral ommatoids absent (Figure 12D,E). Pygidial flagellum comprising at least 25 segments (some may be missing); each segment with numerous macrosetae, without ventromedian ommatoids; first segment very long (length five times the maximum width), distinctly longer than distal segment of pygidium (XII), sinuous in lateral aspect, broadening distally (Figure 12E and Figure 13C); other segments short, about one-quarter length of first segment or less.

*Male gonopods*: Chitinized arches and gonopods as in Figure 11B,D. LoD circular, flat, and membranous; Fi small, subtriangular, with sclerotized lateral tips; LoL1 1.7 times broader than long, trapezoidal in shape, membranous, globose, with few sclerotized wrinkles and rounded posterior margin extending beyond posterior margin of chitinized arch; LoL2 membranose, flat, covered by LoL1; Me subcylindrical, short, 1.8 times longer than wide, sclerotized with unsclerotized anteromedian notch; LaM sclerotized, extending posteriorly; Fu and Pi absent. Chitinized arches of LoD and LoL1/LoL2 fused; arches separate, not fused anteromedially but fused posteromedially; chitinized arch of LoD width regular; anterior, lateral, and posterior margins of chitinized arch of LoL1/Lol2 similar in width.

**Distribution.** *Yekuana wanadi* is known only from the type locality, Santa Elena de Uairén, in the state of Bolívar, Venezuela (Figure 2).

## 4. Discussion

The geographical distributions of the South American hypoctonine taxa are better understood in light of the phylogeny. Prior to recognition of three genera, the distribution of *Thelyphonellus* comprised the Amazonian region of Brazil, Guyana, and Venezuela, crossing the Andes to the Pacific region of Colombia [48,52,53,54,65], which is an exceptional range for a taxon with limited vagility, like whip-scorpions. Although some arachnid taxa are capable of overcoming barriers like the Andes due to mechanisms for long-range dispersal, e.g., ballooning [66], this is impossible for thelyphonids.

The revised generic classification of South American hypoctonine taxa based on the phylogenetic hypothesis is more consistent with their disjunct geographical distributions. *Thelyphonellus*, as here redefined, is restricted to the Amazonian region of Guyana, Suriname, French Guiana, and northern Brazil; *Yekuana* occurs in the Guiana Shield of Venezuela; and *Wounaan* occurs in the Andean and Pacific regions of Colombia (Figure 2).

The female genitalia of Neotropical hypoctonine whip-scorpions (e.g., Figure 18 and Figure 19) have provided characteristics at the generic level, for example, in the diagnosis of *Ravilops*, originally created for *R. wetherbeei*, from the Dominican Republic and previously placed in *Thelyphonellus* [52]. Unfortunately, female specimens were only available for two species, *T. amazonicus* and *W. vanegasae*. Characteristics of the female genitalia were omitted from the phylogenetic analysis in order to reduce the number of missing entries. However, it is important to note that there is congruence between the morphology of the female genitalia and the topology obtained by the analysis. For example, the receptacula are short and circular in three species of the clade comprising *R. kovariki* [55] (p. 19: Figure 5B), *R. wetherbeei* (Figure 18A; [52] (p. 98: Figure 3C,D)), *W. vanegasae* (Figure 19D), and *W. yarigui* (known only from the male) (Figure 1) but elongated and subcylindrical in *T. amazonicus* (Figure 18B and Figure 19A,B; [52] (p. 98: Figure 3A)) and *T. ruschii* (Figure 19C; [52] (p. 98: Figure 3B)). Despite the differences, a larger sample is necessary to understand interspecific variation and properly assess homology in these structures.

The hemispermatophore has not previously been used for the classification of Neotropical hypoctonines. Hemispermatophores were unavailable for several of the species examined herein. Among those examined, however, e.g., *T. amazonicus* and *Y. wanadi* (Figure 14), the differences were generally uninformative. A proper assessment of this structure, which will require a comprehensive taxonomic sample across the breadth of the lineage, was beyond the scope of the study.

Prior to the present investigation, no study had assessed the phylogeny of any thelyphonid genera using morphology, despite the fact that existing generic classifications have been based entirely on somatic characteristics (e.g., [47,67]). Weygoldt (1979) [48] originally identified *Thelyphonellus* based on the absence of lateral carinae between the median ocelli and between the median and lateral ocelli, the fine granulation of carapace and tergites, the smooth surfaces of the pedipalps and sternites, the complete division of sternites II and III, the unmodified genital operculum (sternite II), the shape of the female genitalia receptacula, and the presence or absence of small, oval ommatoids. Some of these characteristics are largely congruent with the character system used in the present phylogenetic study and confirmed to be of taxonomic significance, including characteristics associated with the surface macrosculpture of the pedipalp tegument, the shape and subdivision of the tergites, the shape of sternite II (genital), the presence or absence of ommatoids, and so on. Several somatic regions were found to contribute useful characteristics, the most important being the pedipalp (especially of the male) and opisthosoma, which contributed 15 and 13 characteristics, respectively (Appendix C). The inclusion of a broader taxonomic sample representing additional taxa and geographical areas will undoubtedly increase the number of characteristics useful for thelyphonid systematics.

## Data Availability

The morphological characteristic matrix and characteristic list were deposited into MorphoBank (Project 5288: Phylogeny of the Neotropical Hypoctonine Whip-scorpions).

## Appendix A

Comparative material examined for phylogenetic analysis of Neotropical Hypoctoninae Pocock, 1899. Material was deposited in the following collections: the American Museum of Natural History (AMNH), including the Ambrose Monell Cryocollection for Molecular and Microbial Research (AMCC), New York, NY, USA; the Instituto de Investigación de Recursos Biológicos “Alexander von Humboldt” (IAvH), Villa de Leyva, Colombia; the Museo Departamental de Ciencias Naturales “Federico Carlos Lehmann Valencia” (IMCN), Instituto para la Investigación y la Preservación del Patrimonio Cultural y Natural del Valle del Cauca, Cali, Colombia; the Museu de Zoologia, Universidade de São Paulo (MZSP), Brazil; the Museu Paraense Emílio Goeldi (MPEG), Belém, Brazil; the Museum für Naturkunde der Humboldt-Universität, Berlin, Germany (ZMB); the Museum of Comparative Zoology (MCZ), Harvard University, Cambridge, MA, USA; the Natural History Museum, London, UK (BMNH); the Senckenberg Forschungsinstitut und Naturmuseum, Frankfurt (SMF), Germany; and the USA. National Museum of Natural History (USNM), Smithsonian Institution, Washington, DC, USA.

*Etienneus africanus* (Hentschel, 1899): **THE GAMBIA:** *Central River*: Sapu Agricultural Station, 180 mi. upriver [13°33′ N 14°54′ W], 1968, G.C.H. Smith, 1 juv. (AMNH IZC 325051). **GUINEA-BISSAU:** *Bafatá Region*: 19 km S of Bambadinca, 11°53′03.9″ N 14°50′08.5″ W, 38 m, 30.vi–1.vii.2005, J. Huff and V. Vignoli, wet savannah, with marginal secondary rainforest, hand-collected under rocks and logs, 1 ♀, 1 juv. (AMCC [LP 4654]), 1 subad. ♀, 2 juv. (AMNH IZC 325032). **SENEGAL:** *Région de Tambacounda*: Kedougou, 3 km W on road to Salemata, 12°33′10.6″ N 12°13′39.4″ W, 126 m, 4.vii.2005, J. Huff and V. Vignoli, sparse forest with small boulders, hand-collected under rocks and logs, 1 ♂, 1 ♀ (AMNH IZC 357162).

*Ginosigma schimkewitschi* (Tarnani, 1894): **VIETNAM:** *Kiên Giang Province*: Kiên Lương District, Hòn Chồng, forest trail, 10°09′17.8” S 10°09′27.3” S to 104°37′33.2” E 104°37′21.98” E, 21–41 m, 3.vi.2008, R.W. Murphy, J, Che, and N. Ngoc Sang, 11 ♂, 1 ♀ (AMNH IZC 325033).

*Ravilops kovariki* Teruel, 2017: **DOMINICAN REPUBLIC:** *Bahoruco Province*: Parque Nacional Sierra de Neyba, Los Guineos, 12.4 km N of Neyba, 18°35′17.3″ N 71°25′47.8″ W, 742 m, 17.vii.2010, J. Huff and A. Sanchez, small river gorge with coffee and banana plantation, 1 subad. ♂, 1 juv. ♀ (AMCC [LP 10499]).

*Ravilops wetherbeei* (Armas, 2002): **DOMINICAN REPUBLIC:** *Santiago Province*: Parque Nacional Armando Bermúdes, Mata Grande Station, 19°12′07.7″ N 70°59′59.3″ W, 975 m, 22.vii.2004, E.S. Volschenk and J. Huff, rainforest with breaks of bracken, hand-collected from under stones and logs, 1 ♀ (AMCC [LP 3342]); along road to Mata Grande Station, 19°13′19.2″ N 70°57′26.6″ W, 914 m, 22.vii.2004, E.S. Volschenk and J. Huff, hand-collected from under stones and logs, 2 ♂ (AMNH IZC 326332), 19°13′18.84″ N 70°57′27″ W, 914 m, 22.vii.2004, E.S. Volschenk and J. Huff, hand-collected from under stones and logs, 1 juv. ([AMCC [LP 3343]).

*Thelyphonellus amazonicus* (Butler, 1972): **BRAZIL:** *Amapá*: Serra do Navio [00°54′ N 52°00′ W], 13.i.1962, C. Froehlich, 1 ♂ (MZSP 28328), 13.i.1962, C. Froehlich and Narchi, 1 ♂ (MZSP 14319), 13–14.v.1992, G. Skuk, 1 ♂ (MZSP 14315); Fazenda Campo Verde, near Pôrto Platon [00°42′ N 51°26′ W to 00°54′ N 51°37′ W], 12.i.1962, C. Froehlich, 1 ♂ (MZSP 14320). *Amazonas*: 24 km NE of Manaus [02°44′S 59°53′ W], Manaus to Rio Branco road at km 5, 26.viii.1962, W.L. Brown, 1 ♀ (MCZ 64457); Manaus [03°07′S 60°01′ W], 4.ix.1962, K. Lenhow, 1 ♀ (MZSP 14316), 27.viii.1962, K. Lenhow, 1 juv. (MZSP 14318), 7.ix.1962, K. Lenhow, 1 juv. (MZSP 14317). *Pará*: Almeirim, Jari Celulose S.A., 00°41′25.94”S 52°49′09.22″ W, 17–23.viii.2004, T. Gardner, 1 ♂ (MPEG [URO] 5), 1 ♀ (MPEG [URO] 1), 00°42′33.19” S 52°46′57.49″ W, 23.viii.2004, T. Gardner, 3 ♂, 1 ♀ (MPEG [URO] 3), 00°42′42.65” S 52°40′00.86″ W, 11.ii.2005, T. Gardner and M.A. Ribeiro Júnior, 2 ♂ (MPEG [URO] 4, 11), 11.ii.2005, T. Gardner, 2 ♂ (MPEG [URO] 6, 8), 1 ♀ (MPEG [URO] 13), 01°01′32.46” S 52°54′17.28″ W, 3.iv.2005, T. Gardner and M.A. Ribeiro Júnior, 1 ♀ (MPEG [URO] 10), 01°01′33.12” S 52°34′02.78″ W, 22.vi.2005, T. Gardner and M.A. Ribeiro Júnior, 4 ♂ (MPEG [URO] 2, 9, 12), 1 ♀ (MPEG [URO] 7), 01°11′28.31” S 52°38′51.86″ W, 28.viii–3.ix.2004, T. Gardner, 1 ♂, 1 subad. ♀ (MPEG [URO] 14); Juruti, Sítio Barroso, 02°27′41.7” S 56°00′11.6″ W, 8.viii.2006, D.F. Candiani and N.F. Lo-Man-Hung, 1 juv. (MPEG [URO] 15), 10.viii.2006, Equipe Herpetologia, 1 ♂ (MPEG [URO] 40), 11.viii.2006, D.F. Candiani, 1 ♂ (MPEG [URO] 23), 15.viii.2006, Equipe Herpetologia, 1 juv. (MPEG [URO] 26), 7.vi.2007, D.F. Candiani, 1 ♀ (MPEG [URO] 45), 22.xi.2007, D.F. Candiani and N.F. Lo-Man-Hung, 1 ♀ (MPEG [URO] 31); Juruti, Platô Capiranga, Linha 168E, 02°28′22.1” S 56°12′29.4″ W, 15.viii.2006, D.F. Candiani and N.F. Lo-Man-Hung, 1 juv. (MPEG [URO] 18), 10.ii.2007, J.A.P. Barreiros, 1 ♂ (MPEG [URO] 44), 13.ii.2007, N.F. Lo-Man-Hung and J.A.P. Barreiros, 1 ♀ (MPEG [URO] 36), 15.ii.2007, N.F. Lo-Man-Hung and J.A.P. Barreiros, 2 ♂ (MPEG [URO] 42), 8.vi.2007, N.F. Lo-Man-Hung, 1 ♀ (MPEG [URO] 38), 19.x.2007, A. Lima and F. Pimenta, 1 ♂ (MPEG [URO] 39), 22.xi.2007, N.F. Lo-Man-Hung and D.F. Candiani, 1 juv. (MPEG [URO] 17); Juruti, Ramal Pacoval, km 2, 02°28′00.6″ S 56°12′42.2″ W, 21.xi.2007, D.F. Candiani, 2 ♂ (MPEG [URO] 35); Juruti, Sítio Três Irmãos, 02°27′51.4″ S 56°00′08.6″ W, 3.iii.2006, 1 ♂ (MPEG [URO] 20), 7.iii.2006, D.R. Santos-Souza, 3 ♂ (MPEG [URO] 19), 8–15.viii.2006, D.F. Candiani and N.F. Lo-Man-Hung, 1 juv. (MPEG [URO] 41), 15.viii.2006, D.F. Candiani and N.F. Lo-Man-Hung, 1 juv. (MPEG [URO] 46), 8.ii.2007, J.A.P. Barreiros, 1 ♂ (MPEG [URO] 22), 8.ii.2007, N.F. Lo-Man-Hung, 1 ♂ (MPEG [URO] 33), 14.ii.2007, A. Lima, P. Suarez and S. Abrantes, 1 ♂ (MPEG [URO] 28), 15.ii.2007, N.F. Lo-Man-Hung and J.A.P. Barreiros, 1 ♂ (MPEG [URO] 43), 16.xi.2007, D.F. Candiani, 1 ♀ (MPEG [URO] 34); Juruti, Vale do Igarapé Mutum, Platô do Rio Juruti, 02°36′45.7″ S 56°11′38.2″ W, 15.viii.2006, D.F. Candiani and N.F. Lo-Man-Hung, 1 subad. ♀ (MPEG [URO] 27), 2 juv. (MPEG [URO] 16, 25), 20.xi.2007, N.F. Lo-Man-Hung, 1 ♂ (MPEG [URO] 37); Juruti, Vale do Igarapé Mutum, Platô do Rio Juruti, 01°36′44.7″ S 56°11′39.2″ W, 10.viii.2006, D.F. Candiani, 1 ♂ (MPEG [URO] 32), 13.ii.2007, N.F. Lo-Man-Hung and J.A.P. Barreiros, 1 juv. (MPEG [URO] 29), 10.vi.2007, D.F. Candiani and N.F. Lo-Man-Hung, 3 ♂ (MPEG [URO] 21, 24, 30); Melgaço, Estação Científica Ferreira Penna, FLONA Caxiuanã, IMC 2a expedição, 01°44′18.02″ S 51°27′48.01″ W, 14.x–16.xi.2003, J.A.P. Barreiros, 1 juv. (MPEG [URO] 47), Flona Caxiuanã, 14.x–16.xi.2003, J.A.P. Barreiros, 1 ♀ (MPEG [URO] 48); Santarém, Fazenda Taperinha, xi.1970, EPA, 1 ♀ (MZSP 14314). **FRENCH GUIANA:** *Roura*: Cacao-Sentier Molokoi, 04°33′41.1″ N 52°27′44″ W, 95 m, 19.xii.2004, J. Huff, hand-collected under rocks and wood in primary tropical rainforest, 1 ♀ (AMCC [LP 3837]). *St. Georges-Oyapok*: SW of St. Georges on road to Maripa, 03°50′17.3″ N 51°52′19″ W, 53 m, 18.xii.2004, J. Huff, hand-collected under wood in primary tropical rainforest, 1 ♂ (AMCC [LP 3835]). **SURINAME:** 2.ii.1942, L. Schmidt, 1 ♂ (MCZ 64454). *Sipaliwini*: Brownsberg Natural Reserve [04°56′ N 55°10′ W], 5.ix.1977, R.T. Sawyer, 1 juv. (USNM 2050707).

*Thelyphonellus* aff. *ruschii*: **GUYANA:** *Potaro-Suparuni Region*: Iwokrama Forest Reserve, Turtle Mountain, trail from camp to Turtle Mountain Lookout, 04°43′54.85″ N 58°43′06.58″ W to 04°43′57.35″ N 58°44′01.17″ W, 73–274 m, N. Cazzaniga, P. Colmenares and L. Prendini, 10.ix.2023, primary rainforest at base, slopes, and summit of low hill with rocky granite outcrops near summit; dense canopy, sparse understorey, moderate to dense litter layer, clayey-loam soil; flashlight on warm, dark, humid night; light breeze near hill summit, running on path in rocky area, 1 ♂ (AMNH IZC 357759).

*Wounaan vanegasae* (Giupponi and Vasconcelos, 2008), **comb. n.**: **COLOMBIA:** *Valle del Cauca Department*: 28 km E of Buenaventura [03°51′ N 76°49′ W], 50 m, ii.1970, 1 ♂ (MCZ 64455); near Queremal [03°31′ N 76°42′ W], 74 km on new road Cali–Buenaventura, 12–13.ii.1976, under rock, 1 juv. (MCZ 64451); San Francisco, Buenaventura [03°52′ N 77°02′ W], xii.1988, 80 m, E. Flórez, 1 ♀ (IMCN 9931).

*Wounaan yarigui*, **gen. et sp. n.**: **COLOMBIA:** *Santander Department*: Carmen de Chucurí, Vereda La Belleza, 06°34′13″ N 73°34′15″ W, 844 m, 22.ii.2018, J.C. Neita, E. Torres, and M.I. Castro, tropical humid forest, pitfall, holotype ♂ (IAvH I 2831).

*Yekuana venezolensis* (Haupt, 2009), **comb. n.**: **VENEZUELA:** *Edo. Bolívar*: San Isidro [06°09′ N 61°25′ W], 88 km on road to Guiana upland (southern periphery of San Isidro), iii.2008, V. Šejna, holotype ♂, 1 subad. ♂ paratype (ZMB 48289).

*Yekuana wanadi*, **gen. et sp. n.**: **VENEZUELA:** *Edo. Bolívar*: St. Elena de Uairén [Santa Elena de Uairén, 04°36′23.2″ N 61°06′19.4″ W], km 315, 14.xi.2005–ii.2006, C. Seiderman, holotype ♂ (AMNH IZC 325050).

*Yekuana* sp. **VENEZUELA:** *Bolívar*: Sifontes, La Escalera, between Piedra de la Virgen and Monumento Soldado Pionero, 06°04′12.66″ N 61°23′50.16″ W, 523 m, 16.vii.2009, A. Yepes, M. Blanco and J.A. Ochoa, 1 ♂ (AMCC [LP 10075]).

## Appendix B

Distribution of 45 morphological characteristics used for phylogenetic analysis of Neotropical Hypoctoninae Pocock, 1899. Character states scored 0–5 or unknown (?). First taxon is outgroup.

*Ginosigma schimkewitschi* (Tarnani, 1894) 00011 11010 01102 21130 00001 10110 00010 00101 13120 *Etienneus africanus* (Hentschel, 1899) 02000 00001 12220 00000 00000 00001 11100 100[01]0 03000 *Ravilops kovariki* Teruel, 2017 ?3?0? 00?1? 1?0?2 3?12? ?1?00 0???? ???00 ?0010 02000 *Ravilops wetherbeei* (Armas, 2002) 12100 00010 11012 33121 01000 01000 11500 00010 02010 *Thelyphonellus amazonicus* (Butler, 1972) 12101 00010 12002 02110 00000 00000 11401 02010 01020 *Thelyphonellus* aff. *ruschii* 12101 00010 12002 02110 00000 00?0? 11401 020?0 01020 *Wounaan vanegasae* (Giupponi and Vasconcelos, 2008), **comb. n.** 12100 00110 12031 13221 11101 01000 11200 00010 02000 *Wounaan yarigui*, **gen. et sp. n.** 11100 00110 12031 13221 11111 01?0? 11200 000?0 02000 *Yekuana venezolensis* (Haupt, 2009), **comb. n.** 13101 00010 10012 22110 00000 10?0? 11300 010?0 00000 *Yekuana wanadi*, **gen. et sp. n.** 13101 00010 10012 12110 00000 10?0? 11300 010?0 00031

## Appendix C

Morphological characteristics used for phylogenetic analysis of Neotropical Hypoctoninae Pocock, 1899. Character statements apply to both male and female unless specified otherwise. Abbreviations: L, length; CI, consistency index; RI, retention index; †, uninformative.

**Carapace**

1. Carapace anterior margin, surface macrosculpture and setation (L = 1, CI: 1, RI: 1): (0) serrate (conspicuously granular) and densely setose (row of ca. eight macrosetae intercalated with granules anterior to median ocelli); (1) smooth and sparsely setose. The number of macrosetae in the South American species is unclear but appears to be four or fewer.
2. Carapace anterior margin, shape (♂) (L: 4, CI: 0.75, RI: 0.5): (0) truncate (sublinear); (1) semi-elliptical; (2) slightly pointed; (3) markedly pointed. The carapace anterior margin is markedly pointed in *Ravilops kovariki* Teruel, 2017, *Yekuana wanadi*, **gen. et sp. n.**, and even more so in *Yekuana venezolensis* (Haupt, 2009), **comb. n.**
3. Carapace anterior third, W-shaped smooth surface (L: 1, CI: 1, RI: 1): (0) absent, surface between median and lateral ocelli irregular like rest of carapace; (1) present and distinct.
4. Carapace sulci, development (L: 1, †): (0) weak; (1) pronounced.
5. Carapace fovea, shape and development (♂) (L: 2, CI: 0.5, RI: 0.66): (0) short, aligned with leg III trochanter, very shallow (barely visible) to moderately shallow (distinct); (1) elongated, aligned with leg III trochanter and slightly extending beyond it anteriorly, deep or moderate. The short fovea is very shallow in *Etienneus africanus* (Hentschel, 1899), *Ravilops wetherbeei* (Armas, 2002), and *Wounaan yarigui*, **gen. et sp. n.** and moderately shallow in *Wounaan vanegasae* (Giupponi and Vasconcelos, 2008), **comb. n.**, whereas the long fovea is deep in *Ginosigma schimkewitschi* (Tarnani, 1894), *Y. venezolensis*, and *Y. wanadi* and moderate in *Thelyphonellus amazonicus* (Butler, 1972). This characteristic was scored for the male only, as some species may possess an intersexual variation not yet discovered.
6. Carapace anterolateral oblique carinae (L: 1, †): (0) absent; (1) present, pronounced.
7. Carapace median ocular tubercle, superciliary carina (separating ocelli) (L: 1, †): (0) absent or indistinct; (1) present, pronounced. Absent or indistinct in *Thelyphonellus* aff. *ruschii* and presumed to also be absent or indistinct in *Thelyphonellus ruschii* Weygoldt, 1979, as there was no mention of this conspicuous structure by Weygoldt (1979) [48].
8. Carapace anteromedian longitudinal raised surface (anterior to median ocular surface, different to superciliary carina) (L: 1, CI: 1, RI: 1): (0) absent, anteromedian surface not raised; (1) raised surface present, moderate (not obscuring anteromedian epistome in dorsal aspect), or pronounced (obscuring anteromedian epistome in dorsal aspect). The raised surface is moderately developed in *W. vanegasae* and pronounced in *W. yarigui*.
9. Carapace lateral ocular tubercle, number of central (minute) ocelli (L: 1, †): (0) one ocellus (anterodorsal); (1) two ocelli (anterodorsal and posteroventral).

**Sternum**

10. Anterior sternum (prosternum), median longitudinal suture (L: 1, †): (0) absent, prosternum entire; (1) present, prosternum divided.
11. Anterior sternum (prosternum), posterior stylet-like section, shape (L: 1, †): (0) very narrow and needle-shaped, partially obscured by abutting coxae II; (1) relatively broad and arrow-shaped, completely exposed (not obscured by coxae II).
12. Median sternum (mesosternum) sclerotization (L: 3, CI: 0.66, RI: 0.5): (0) markedly sclerotized and pigmented across entirety, entire (not divided longitudinally); (1) markedly sclerotized and pigmented anteriorly only, rest of mesosternum pale and depigmented; (2) two markedly sclerotized and pigmented areas, anteriorly and posteriorly, separated by pale, depigmented area medially, posterior pigmented area longitudinally divided or entire (undivided). The posterior pigmented area is entire in *W. vanegasae* but longitudinally divided in *E. africanus*, *T. amazonicus*, and *W. yarigui*.

**Pedipalps**

13. Pedipalp dorsal, ventral, and retrolateral surfaces, surface macrosculpture (L: 2, †): (0) predominantly smooth, shiny; (1) trochanter and femur markedly irregular and punctate, especially dorsally; (2) trochanter, femur, and to a lesser extent patella and tibia coarsely granular, especially on dorsal and ventral surfaces.
14. Pedipalp cuticle, microsculpture (L: 3, CI: 1, RI: 1): (0) dorsal and retrolateral surfaces of segments entirely smooth (except for punctations, addressed in character 13, in *G. schimkewitschi*); (1) dorsal and retrolateral surfaces of segments entirely smooth, except for chela fingers with minute reticulation (visible at great magnification); (2) dorsal, retrolateral, ventral, and prolateral surfaces of segments predominantly smooth (except for coarse granules, addressed in character 13) but with fine yet distinct reticulation (visible at great magnification); (3) dorsal and retrolateral surfaces of segments predominantly smooth but with fine yet distinct reticulation (visible at great magnification). Chela retrolateral surface and to a lesser extent dorsal surface distinctly scabrose across extensive area in *W. yarigui*.
15. Pedipalp trochanter, principal (fourth) prodorsal tubercle, development (♂) (L: 2, CI: 1, RI: 1): (0) pronounced, others greatly reduced or granuliform; (1) similar to or shorter than adjacent (third and fifth) tubercles; (2) larger than other tubercles.
16. Pedipalp trochanter, proventral distal tubercle, development (♂) (L: 4, CI: 0.75, RI: 0.75): (0) small, not enlarged; (1) moderate (about as long as broad); (2) slightly enlarged (slightly longer than broad); (3) markedly enlarged (much longer than broad).
17. Pedipalp femur, proventral tubercle, development (♂) (L: 3, CI: 1, RI: 1): (0) absent or greatly reduced (undifferentiated from adjacent coarse granules); (1) small, granuliform; (2) moderate, subspiniform; (3) large, spiniform.
18. Pedipalp patellar apophysis, length relative to patella width (♂) (L: 2, CI: 1, RI: 1): (0) short, length several times less than patella width; (1) moderate, length slightly less than patella width; (2) long, length greater than patella width.
19. Pedipalp patellar apophysis, number of granules on prolateral (anterior) margin (♂) (L: 3, CI: 1, RI: 1): (0) only one basal granule; (1) row of 3–5 granules (not including apex); (2) row of 6–9 granules (not including apex); (3) row of 12 or 13 granules (not including apex). Row of 3–4/3–4 granules in *Y. venezolensis*, 4–5 granules in *T. amazonicus*, 5/5 granules in *T.* aff. *ruschii* and *Y. wanadi*, 6–8 granules in *R. wetherbeei*, 7/8 granules in *W. yarigui*, 8/9 granules in *W. vanegasae*, and 9 granules in *R. kovariki*; 5 granules estimated in *T. ruschii*, based on Weygoldt (1979) [48].
20. Pedipalp patella, proventral distal tubercle, development (♂) (L: 1, CI: 1, RI: 1): (0) small or obsolete; (1) moderate, distinct.
21. Pedipalp tibia (manus), shape (♂) (L: 1, CI: 1, RI: 1): (0) unmodified, not dorsoventrally expanded (barrel-shaped); (1) markedly expanded dorsoventrally (subcircular in lateral aspect, not barrel-shaped). The manus of *E. africanus* is swollen yet barrel-shaped and unmodified in shape.
22. Pedipalp tibia (manus), proventral distal tubercle, development (♂) (L: 1, CI: 1, RI: 1): (0) small, rounded or subtriangular; (1) large, spiniform. The proventral distal tubercle is rounded in *Y. wanadi*, *Y. venezolensis*, and *G. schimkewitschi* (obsolete in the latter) and subtriangular in *E. africanus* and rounded or subtriangular in *T. amazonicus*.
23. Pedipalp tibia (manus), ventral part of retrolateral surface (i.e., retrolateral surface aligned with movable finger), surface (♂) (L: 1, CI: 1, RI: 1): (0) unmodified, slightly convex like rest of retrolateral surface; (1) planar or concave. The ventral part of the surface is planar in *W. vanegasae* but noticeably concave in *W. yarigui*.
24. Pedipalp fixed (tibial) finger, retrolateral surface (♂) (L: 1, †): (0) unmodified, slightly convex (like retrolateral surface of manus); (1) planar, flat. Presumably unmodified in *R. kovariki*, based on Teruel (2017) [55].
25. Pedipalp fixed (tibial) finger, ventral row of denticles, shape in retrolateral aspect (♂) (L: 2, CI: 0.5, RI: 0.5): (0) linear; (1) slightly or markedly sinuous. The row is slightly sinuous in *G. schimkewitschi* and *W. vanegasae* but markedly sinuous in *W. yarigui*.
26. Pedipalp movable finger (tarsus), dorsal row of denticles, basal lobe (♂) (L: 2, CI: 0.5, RI: 0.5): (0) absent or obsolete; (1) pronounced. Presumably absent or obsolete in *R. kovariki*, based on Teruel (2017) [55].
27. Pedipalp movable finger (tarsus), dorsal row of denticles, distal lobe (♂) (L: 1, CI: 1, RI: 1): (0) absent; (1) distinct, shallow, or small. The distal lobe is likely produced by a subtle median emargination of the denticle row.

**Legs**

28. Leg I, seventh to ninth tarsomeres, shape (♀) (L: 1, †): (0) unmodified, seventh and eighth tarsomeres similar to others and all three tarsomeres similar to same tarsomeres of male; (1) modified, with projections and bumps. The ninth tarsomere of the female bears an apical, subconical projection in *E. africanus* (character 30).
29. Leg I tarsus, apical (ninth) tarsomere, length relative to adjacent tarsomeres (L: 1, †): (0) moderately long, length similar to sum of lengths of preceding two (seventh and eighth) tarsomeres; (1) very long, length similar to sum of preceding three (sixth to eighth, in male) or preceding five or six (third or fourth to eighth, in female) tarsomeres.
30. Leg I tarsus, apical (ninth) tarsomere, shape, apical subconical projection (♀) (L: 1, †): (0) unmodified, apical subconical projection absent; (1) modified, apical subconical projection present.
31. Walking legs (II–IV) telotarsi, ventral macrosetae, structure (L: 1, †): (0) spiniform; (1) setiform.
32. Walking legs (II–IV) telotarsi, ventral macrosetae, arrangement (L: 1, †): (0) two (proventral and retroventral) rows; (1) not arranged in rows.

**Opisthosoma**

33. Opisthosomal tergites, subdivision (♂) (L: 5, CI: 1, RI: 1): (0) I entire, II–IX each with distinct, complete or almost complete median longitudinal suture; (1) I entire, II–IV each with distinct median longitudinal suture, complete, V and VI and to lesser extent VII each with longitudinal suture anteriorly only, other tergites undivided; (2) I entire, II and III each with distinct median longitudinal suture, complete, IV with equally distinct longitudinal suture anteriorly only, other tergites undivided; (3) I entire, II and III each with distinct median longitudinal suture, complete, IV and to lesser extent V each with longitudinal suture anteriorly only (obsolete in both), other tergites undivided; (4) I entire, II and III each with distinct median longitudinal suture, complete, IV and to lesser extent V–VIII each with longitudinal suture anteriorly only (obsolete in all but IV), other tergites undivided; (5) I partially divided (posteriorly only) and terminating in triangular hyaline area, II and III each with distinct median longitudinal suture, complete, IV and to lesser extent V each with longitudinal suture anteriorly only (obsolete in both), other tergites undivided.
34. Opisthosomal tergites II and III, size (i.e., relative to tergite IV of male and to homologous tergites of female) (♂) (L: 1, †): (0) unmodified (e.g., tergites II and III each similar in length to IV); (1) noticeably longer (e.g., tergites II and III each ca. 1.6× length of IV).
35. Opisthosomal tergites II and III, posterior margin (L: 1, CI: 1, RI: 1): (0) unmodified, linear (similar to IV–VIII); (1) slightly emarginate medially (unlike IV–VIII, linear).
36. Opisthosomal pleural membranes, granulation (L: 1, †): (0) markedly sclerotized, elongated granules; (1) inconspicuous, weakly sclerotized granules (same or similar color to pleural membrane).
37. Opisthosomal sternite II (genital), posterior margin shape (♂) (L: 2, CI: 1, RI: 1): (0) moderately expanded (enlarged and lobate) and sinuous posteromedially; (1) moderately expanded (enlarged and lobate) and semicircular along entire margin; (2) markedly expanded (enlarged and lobate) and semicircular along entire margin (significantly larger than in female).
38. Opisthosomal sternite II (genital), subdivision (L: 1, †): (0) entire (without median longitudinal suture); (1) partially divided by median longitudinal suture.
39. Opisthosoma, exposed (i.e., visible) part of sternite III, anteromedian pale area (♀) (L: 1, †): (0) absent or not exposed (covered completely by sternite II); (1) exposed, distinct.
40. Opisthosomal sternite III, posterior margin, median spinoid projection (♂) (L: 1, †): (0) absent; (1) present. Absent in *T.* aff. *ruschii* and presumed to be absent in *T. ruschii* because there was no mention of this conspicuous structure by Weygoldt (1979) [48].
41. Opisthosomal sternite V, medial patch of macrosetae (♂) (L: 1, †): (0) absent; (1) present. Absent in *T.* aff. *ruschii* and presumed to be absent in *T. ruschii* because there was no mention to this conspicuous structure by Weygoldt (1979) [48].
42. Opisthosomal pygidium, segment XII (posterior segment), dorsolateral ommatoids, presence and development (L: 3, CI: 1, RI: 1): (0) absent; (1) obsolete, very small and barely visible; (2) medium-sized and well developed; (3) very large and well developed.
43. Opisthosomal pygidial flagellum, individual segments, ventral translucent area (ventromedian ommatoid) (L: 1, †): (0) absent; (1) present (on each segment).
44. Opisthosomal pygidial flagellum, relative proportions of segments (♂) (L: 4, CI: 0.75, RI: 0.5): (0) all segments moderately elongated (similar to or shorter than posterior segment of pygidium); (1) first segment moderate (slightly shorter than posterior segment of pygidium), others relatively short; (2) first segment long (slightly longer than posterior segment of pygidium), others relatively short; (3) first segment very long (noticeably longer than posterior segment of pygidium), others very short (about one-quarter the length of first segment).
45. Opisthosomal pygidial flagellum, basal (first) segment, shape in lateral aspect (♂) (L: 1, †): (0) unmodified (linear), as narrow as others; (1) sinuous, broadening posteriorly.

## References

[1] Haupt J., Höhne G., Weiske T. (1993). Acetic acid esters, N-hexanol, N-octanol, and capronic acid as ingredients in the defense secretion product of whip scorpions. Boll. Delle Sedute Della Accad. Gioenia Di Sci. Nat. Catania.

[2] Haupt J., Müller F. (2004). New products of defense secretion in South East Asian whip scorpions (Arachnida: Uropygi: Thelyphonida). Z. Für Naturforschung C.

[3] Weygoldt P., Paulus H.F. (1979). Untersuchungen zur Morphologie, Taxonomie und Phylogenie der Chelicerata. I. Morphologische Untersuchungen. Z. Die Zool. Syst. Und Evol..

[4] Weygoldt P., Paulus H.F. (1979). Untersuchungen zur Morphologie, Taxonomie und Phylogenie der Chelicerata. II. Cladogramme und die Entfaltung der Chelicerata. Z. Die Zool. Syst. Und Evol..

[5] Shultz J.W. (1990). Evolutionary morphology and phylogeny of Arachnida. Cladistics.

[6] Shultz J.W. (2007). A phylogenetic analysis of the arachnid orders based on morphological characters. Zool. J. Linn. Soc..

[7] Wheeler W.C., Hayashi C.Y. (1998). The phylogeny of the extant chelicerate orders. Cladistics.

[8] World Uropygi Catalog (2024). Natural History Museum Bern. http://wac.nmbe.ch.

[9] Lucas H., Webb P.B., Berthelot S. (1835). Arachnides. Histoire Naturelle des îles Canaries.

[10] Koch C.L. (1843). Die Arachniden. Getreu nach der Natur Abgebildet und Beschrieben.

[11] Girard C., Marcy G.B., McClellan G.B., United States War Department (1854). Exploration of the Red River of Louisiana, in the Year 1852.

[12] Doleschall C.L. (1857). Bijdrage tot de kennis der arachniden van den Indischen Archipel. Natuurkundig Tijdschr. Voor Ned.-Indië.

[13] Doleschall C.L. (1859). Tweede bijdrage tot de kennis der arachniden van den Indischen Archipel. Acta Soc. Sci. Indo-Neerl..

[14] Wood H.C. (1862). Description of a new species of the genus *Thelyphonus*. Proc. Acad. Nat. Sci. Phila..

[15] Wood H.C. (1863). On the Pedipalpi of North America. J. Acad. Nat. Sci. Phila..

[16] Wood H.C. (1864). Description of new species of North American Pedipalpi. Proc. Acad. Nat. Sci. Phila..

[17] Stoliczka F. (1869). Contribution towards the knowledge of Indian Arachnoidea. J. Asiat. Soc. Bengal.

[18] Stoliczka F. (1873). Notes on the Indian species of *Thelyphonus*. J. Asiat. Soc. Bengal.

[19] Butler A.G. (1872). A monograph of the genus Thelyphonus. Ann. Mag. Nat. Hist. (Ser. 4).

[20] Butler A.G. (1873). A monographic revision of the genus *Phrynus*, with descriptions of four remarkable new species. Ann. Mag. Nat. Hist. (Ser. 4).

[21] Simon E. (1877). Arachnides recueillis aus îles Philippines par MM. G.-A. Baer et Laglaise. Ann. Société Entomol. Fr..

[22] Simon E. (1887). Etudes sur les arachnides de l’Asie méridionale fasaint partie des collections de l’Indian Museum (Calcutta). I. Arachnides recueillis à Tavoy (Tennasserim) par Moti Ram. J. Asiat. Soc. Bengal.

[23] Koch L., Keyserling E. (1885). Die Arachniden Australiens.

[24] Thorell T. (1888). Pedipalpi e scorpioni dell’Arcipelago Malese conservati nel Museo Civico di Storia Naturale di Genova. Ann. Del Mus. Civ. Stor. Nat. Genova.

[25] Oates E.W. (1889). On the species of *Thelyphonus* inhabiting continental India, Burma, and the Malay Peninsula. J. Asiat. Soc. Bengal.

[26] Tarnani J. (1889). Sur les collections de Thelyphonides de quelques Musées russes. Zool. Anz..

[27] Tarnani J. (1894). Quelques nouvelles espèces de Thélyphonides. Zool. Anz..

[28] Pocock R.I. (1894). Notes on the Thelyphonidae contained in the collection of the British Museum. Ann. Mag. Nat. Hist..

[29] Kraepelin K. (1897). Revision der Uropygi Thor. (Thelyphonidae auct.). Abh. Aus Dem Geb. Der Naturwissenschaften Hrsg. Vom Naturwissenschaftlichen Ver. Hambg..

[30] Kraepelin K. (1899). Scorpiones und Pedipalpi. Das Tierreich.

[31] Pocock R.I. (1900). Some new or little-known Thelyphonidae and Solifugae. Ann. Mag. Nat. Hist..

[32] Pocock R.I., Godman F.D.C. (1902). Arachnida: Scorpiones, Pedipalpi, & Solifugae. Biologia Centrali-Americana.

[33] Hirst S. (1911). On a new pedipalp from Burma. Ann. Mag. Nat. Hist..

[34] Hirst S. (1912). Descriptions of new arachnids of the orders Solifugae and Pedipalpi. Ann. Mag. Nat. Hist..

[35] Kraepelin K. (1911). Neue Beiträge zur Systematik der Gliederspinnen. Mitteilungen Aus Dem Naturhistorischen Mus. Hambg..

[36] Gravely F.H. (1912). Notes on Pedipalpi in the collection of the Indian Museum. III. Some new and imperfectly known species of *Hypoctonus*. Rec. Indian Mus..

[37] Gravely F.H. (1916). The evolution and distribution of the Indo-Australian Thelyphonidae, with notes on the distinctive characters of various species. Rec. Indian Mus..

[38] Werner F. (1916). Über einige Skorpione und Gliederspinnen der Naturhistorischen Museums in Wiesbaden. Jahrbüchern Des Nassau. Ver. Naturkunde Wiesb..

[39] Werner F. (1932). Die Skorpione und Pedipalpen der Deutschen Limnologischen Sunda-Expedition. Arch. Hydrobiol. Suppl..

[40] Franganillo B.P. (1931). Excursiones aracnológicas durante el mes de Agosto de 1930. Rev. Belén La Habana.

[41] de Mello-Leitão C. (1931). Pedipalpos do Brasil e algumas notas sobre a ordem. Arq. Do Mus. Nac..

[42] de Mello-Leitão C. (1940). Um pedipalpo e dois escorpiões da Colômbia. Papéis Avulsos Dep. Zool. Secr. Agric. São Paulo.

[43] Speijer E.A.M. (1931). Bemerkungen über Pedipalpi. Zoölogische Meded..

[44] Speijer E.A.M. (1933). [No title]. Tijdschr. Voor Entomol..

[45] Speijer E.A.M. (1936). Die orientalischen Pedipalpi des Zoologischen Museums der Universität Berlin. Mitt. Aus Dem Zool. Mus. Berl..

[46] Pocock R.I. (1899). The geographical distribution of the Arachnida of the orders Pedipalpi & Solifugae. Nat. Sci..

[47] Rowland J.M., Cooke J.A.L. (1973). Systematics of the arachnid order Uropygida (= Thelyphonida). J. Arachnol..

[48] Weygoldt P. (1979). *Thelyphonellus ruschii* n. sp. und die taxonomische Stellung von *Thelyphonellus* Pocock 1894 (Arachnida: Uropygi: Thelyphonida). Senckenberg. Biol..

[49] Haupt J., Song D. (1996). Revision of East Asian whip scorpions (Arachnida Uropygi Thelyphonida). I. China and Japan. Arthropoda Sel..

[50] de Armas L.F. (2000). Los vinagrillos de Cuba (Arachnida: Uropygi: Thelyphonidae). Poeyana.

[51] de Armas L.F. (2002). Nueva especie de *Thelyphonellus* (Thelyphonida: Thelyphonidae) de La Espanola, Antillas Mayores. Rev. Ibérica Aracnol..

[52] Víquez C., de Armas L.F. (2005). Dos nuevos géneros de vinagrillos de Centroamérica y las Antillas (Arachnida: Thelyphonida). Boletín Soc. Entomológica Aragonesa.

[53] Giupponi A.P.d.L., Vasconcelos E.G. (2008). Nova espécie de *Thelyphonellus* Pocock, 1894 da Colombia (Arachnida: Thelyphonida: Thelyphonidae). Rev. Ibérica Aracnol..

[54] Haupt J. (2009). *Thelyphonellus venezolanus* n. sp., a new species of vinegaroons (Arachnida: Uropygi: Thelyphonida). Rev. Ibérica Aracnol..

[55] Teruel R. (2017). Una especie nueva de *Ravilops* Víquez & Armas, 2005 de República Dominicana, Antillas Mayores (Thelyphonida: Thelyphonidae). Rev. Ibérica Aracnol..

[56] Huff J.C., Prendini L. (2009). On the African whip scorpion, *Etienneus africanus* (Hentschel, 1899) (Thelyphonida: Thelyphonidae), with a redescription based on new material from Guinea-Bissau and Senegal. Am. Mus. Novit..

[57] Clouse R.M., Branstetter M.G., Buenavente P., Crowley L.M., Czekanski-Moir J., General D.E.M., Giribet G., Harvey M.S., Janies D.A., Mohagan A.B. (2017). First global molecular phylogeny and biogeographical analysis of two arachnid orders (Schizomida and Uropygi) supports a tropical Pangean origin and mid-Cretaceous diversification. J. Biogeogr..

[58] Seraphim G., Giupponi A.P.L., Miranda G.S. (2019). Taxonomy of the thelyphonid genus *Typopeltis* Pocock, 1894, including homology proposals for the male gonopod structures (Arachnida, Thelyphonida, Typopeltinae). ZooKeys.

[59] Barrales-Alcalá D., Francke O.F., Prendini L. (2018). Systematic revision of the giant vinegaroons of the *Mastigoproctus giganteus* complex (Thelyphonida: Thelyphonidae) of North America. Bull. Am. Mus. Nat. Hist..

[60] Goloboff P.A., Farris J.S., Nixon K.C. (2008). TNT, a free program for phylogenetic analysis. Cladistics.

[61] Goloboff P.A., Catalano S.A. (2016). TNT version 1.5, including a full implementation of phylogenetic morphometrics. Cladistics.

[62] Goodman M., Olson C.B., Beeber J.E., Czelusniak J. (1982). New perspectives in the molecular biological analysis of mammalian phylogeny. Acta Zool. Fenn..

[63] Bremer K. (1988). The limits of amino acid sequence data in angiosperm phylogenetic reconstruction. Evolution.

[64] Nixon K. (2002). WinClada.

[65] Harvey M.S. (2003). Order Uropygi. Catalogue of the Smaller Arachnid Orders of The World. Amblypygi, Uropygi, Schizomida, Palpigradi, Ricinulei and Solifugae.

[66] Salgado-Roa F.C., Pardo-Diaz C., Rueda-M N., Cisneros-Heredia D.F., Lasso E., Salazar C. (2024). The Andes as a semi-permeable geographical barrier: Genetic connectivity between structured populations in a widespread spider. Mol. Ecol..

[67] Rowland J.M. (2002). Review of the South American whip scorpions (Thelyphonida: Arachnida). Amazoniana.

